# Implementation, Experiences, Impact, and Costs of Artificial Intelligence in Chest Diagnostics: Protocol for a Mixed Methods Evaluation

**DOI:** 10.2196/81421

**Published:** 2025-10-31

**Authors:** Angus I G Ramsay, Chris Sherlaw-Johnson, Kevin Herbert, Stuti Bagri, Malina Bodea, Nadia Crellin, Holly Elphinstone, Amanda Halliday, Nina Hemmings, Rachel Lawrence, Cyril Lobont, Pei Li Ng, Joanne Lloyd, Efthalia Massou, Raj Mehta, Stephen Morris, Jenny Shand, Holly Walton, Naomi J Fulop

**Affiliations:** 1 Department of Behavioural Science and Health Institute of Epidemiology and Healthcare University College London London United Kingdom; 2 Nuffield Trust London United Kingdom; 3 Department of Public Health and Primary Care University of Cambridge Cambridge United Kingdom; 4 Public Contributor Cambridgeshire United Kingdom; 5 Public Contributor Devon United Kingdom; 6 Public Contributor London United Kingdom

**Keywords:** artificial intelligence, diagnostic imaging, lung cancer, mixed methods evaluation, patient outcomes, cost-effectiveness, patient experience

## Abstract

**Background:**

The ability to perform complex tasks has seen artificial intelligence (AI) used to support radiology in clinical settings, including lung cancer detection and diagnosis. Evidence suggests that AI can contribute to accurate diagnosis, reduce errors, and improve efficiency. The National Health Service England (NHSE)–funded Artificial Intelligence Diagnostic Fund (AIDF) is currently supporting 12 National Health Service (NHS) networks to implement AI for chest diagnostic imaging. There is, however, limited evidence on real-world AI implementation and use, including staff, patient, and caregiver experience, and costs and cost-effectiveness. A National Institute for Health and Care Research Rapid Service Evaluation Team Phase 1 evaluation provided insights into the early implementation of these tools and developed a framework for monitoring and evaluation of AI tools for chest diagnostic imaging in practice.

**Objective:**

This mixed methods evaluation of AI tools for chest diagnostic imaging aims to address previous research gaps by exploring the implementation of AI tools for chest diagnostic imaging, the impact and costs of implementing these service models, and the experiences of patients, caregivers, and staff.

**Methods:**

This study will be a mixed method evaluation of implementation, experiences, impact, and costs of AI for chest diagnostic imaging in NHS services in England, with the evaluation informed by the Major System Change Framework. Trust-level case studies (3 in-depth and up to 9 light-touch) will be performed, including staff member, patient, and caregiver; NHSE AIDF team interviews; meeting observations; and analysis of key relevant documentation. Qualitative data will be analyzed using Rapid Assessment Procedures and inductive thematic analysis, supplemented by in-depth deductive thematic analysis. Data from case study sites and other relevant sources will be used to assess outcomes at the other sites and for comparators. A pragmatic economic model of the chest diagnostic imaging pathway will be developed to estimate key costs and resource use associated with AI tool deployment. Together with input from national stakeholders and staff workshops, the study findings will then be finalized for reporting.

**Results:**

As of September 2025, trust-level research and development approvals with participating sites are complete, and data collection has commenced. Results are expected to be reported by the end of February 2026.

**Conclusions:**

The study will provide new insights into the facilitators and barriers to the adoption of AI technology in health care and the perceptions of both the general public and health care staff on its use. It will also inform best practices in approaches for service performance evaluation, for the implementation of AI into existing care pathways, and for the development of models to best support evidence-based decision-making. It will thus establish a framework upon which the greatest benefits of the use of AI in health care can be realized.

**International Registered Report Identifier (IRRID):**

DERR1-10.2196/81421

## Introduction

### Background and Rationale

In recent years, UK health care policy and guidance [1-10] and international research have highlighted the potential for artificial intelligence (AI)—advanced technology that can perform complex tasks associated with human intelligence [11-14]—to support and transform health care in areas such as radiology. Research has indicated potential benefits regarding a range of outcomes (eg, detection accuracy, error reduction or prevention, efficiency, decision-making, and reducing workforce burden) [13,15,16]. However, there is mixed evidence for some outcomes (eg, diagnostic accuracy) [17].

UK health policy increasingly recognizes the potential value of AI in supporting the efficiency and effectiveness of services provided by the National Health Service (NHS, the publicly funded health care system). In June 2023, NHS England (NHSE, a public body that runs and oversees the running of the NHS in England) announced the Artificial Intelligence Diagnostic Fund (AIDF), which has invested £21 million (US $27.93 million) to accelerate the deployment and implementation of AI diagnostic tools [18]. The fund focuses on chest x-ray (CXR) and chest computed tomography (CT) scans to improve the diagnosis of lung cancer and other conditions [18] and potentially help to address the current unmet need for faster CXR reporting [18]. In the longer term, the NHS proposes that using AI to assist with the early detection of lung cancer may improve patient care and outcomes [18]. However, guidance from the National Institute for Health and Care Excellence (a public body that assesses cost-effectiveness of health care interventions and provides guidance and recommendations on use of these interventions) has identified numerous evidence gaps that must be addressed in relation to AI for chest diagnostic imaging, including gaps on time saving and resource use, adverse effects, performance in different patient groups, ease of use, and impact [1].

In October 2023, the National Institute for Health and Care Research (NIHR) Rapid Service Evaluation Team (RSET) was commissioned to conduct a rapid evaluation of the implementation and outcomes of AIDF. Phase 1 aimed to establish existing evidence for implementing AI in radiology diagnostics, analyze early implementation of AIDF, and identify approaches for future evaluations of changes of this kind [19]. It comprised a systematic scoping review and an empirical study of network-led procurement of AI tools for chest diagnostic imaging, early deployment of AI tools within NHS hospital organizations; empirical work drew on qualitative, quantitative, and health economic perspectives [19]. The systematic scoping review identified potential benefits of AI in chest diagnostic imaging and radiology more broadly [20-22]. It also identified important gaps in knowledge, especially around real-world implementation, including how AI tools are procured, processes to support preparation for deployment, how staff, patients, and caregivers experience and perceive AI for diagnostics, and impact on service effectiveness and resources; this was partly because there are very few studies of real-world implementation of AI tools for radiology diagnostics [20-22]. The empirical study focused on procurement and early deployment of AI tools for chest diagnostics through AIDF [20,22,23]. It established that these processes took longer than anticipated by national leadership. Findings suggested that procurement and preparation for deployment were complex sociotechnical processes, placing significant demands on already busy staff. Key obstacles included variations in local imaging technology, extensive governance processes, and variable local data infrastructure, while important facilitators included advice and support from program leadership, networks sharing expertise and capacity, commitment from hospital staff and AI suppliers, and dedicated project management [20,22,23]. Phase 1 also delivered insights on implications for future evaluations, identifying several data sources that may support measurement of effectiveness and cost-effectiveness within the English NHS, but also noted that limitations to data availability need to be addressed [20,22].

Importantly, phase 1 was unable to evaluate the impact of AI on clinical practice, patient care and outcomes, and cost-effectiveness, and the perspectives of patients, caregivers, and those in the wider care pathway [20,22,24].

The implementation of AI for chest diagnostic imaging is currently operating across 12 imaging networks of NHS trusts (organizations that provide a range of health care to local populations). Deploying AI tools in this way has the potential to change organization and delivery of care both within and across these organizations, with the aim of improving service delivery and outcomes at regional levels. Therefore, these programs may be conceptualized as examples of “major system change,” defined as “a coordinated, systemwide change affecting multiple organizations and care providers, with the goal of significant improvements in the efficiency of health care delivery, the quality of patient care, and population-level patient outcomes” ([25], p422). The framework of Major System Change of Fulop et al [26] outlines that it is necessary to evaluate all stages of implementation, including the decision to change, the decision on which model to implement, the implementation approach used, the implementation outcomes, and the intervention outcomes. This framework has been used to evaluate several national system changes within the English health care system (eg, reconfiguration of stroke services [26,27], specialist cancer services [28], COVID-19 remote home monitoring [29,30], and prenatal exome sequencing [31]).

While research has shown that AI diagnostic tools have the potential to support and improve the detection of lung cancer, little is known about how effective and cost-effective these tools are, or how staff, patients, and caregivers experience them. For recommendations to be made regarding the implementation of AI diagnostic tools, these knowledge gaps need to be addressed [4].

### Aims and Objectives

This mixed methods evaluation of AI tools for chest diagnostic imaging aims to address previous research gaps by exploring the implementation of AI tools for chest diagnostic imaging, the impact and costs of implementing these models, and the experiences of patients, caregivers, and staff.

### Research Questions

Table 1 provides details of our research questions (RQs) and which workstreams will address them.

**Table 1:** Workstreams and research questions.

WS^a^ and research question		Subquestions
**1**		
	RQ1^b^. How has AI^c^ for chest diagnostic imaging been implemented and used in practice in England?	How AI is being used—what are the key functions, where it is being used in the diagnostic care pathway? How are staff (clinicians, managers, and administrators) involved in using AI? How did early implementation work, for example, in terms of planning and facilitation? How are patients informed about the use of AI, for example, in terms of communication or consent? How is AI for chest diagnostic imaging being governed, for example, in terms of information and safety? How have relationships with or between services, networks, and suppliers influenced the organization and delivery of AI? Have there been any adaptations in the service model, associated services along the care pathway, or governance over time? To what extent was AI for chest diagnostic imaging implemented, for example, in terms of uptake, spread, and fidelity? How did implementation approaches (eg, leadership, planning, and facilitation) and service models influence implementation outcomes (eg, uptake, spread, and fidelity) What are the implications for equity, diversity, and inclusion? Have there been any unintended consequences of implementing AI? What are the implications for sustainability? Which factors have been influential for implementation, for example, functions of AI, patient groups, organizational context, network leadership, national program or policy?
	RQ2. What are the experiences of staff involved in delivering care supported by AI tools in chest diagnostic imaging?	What are staff experiences of using AI for chest diagnostic imaging? What are the factors (barriers or facilitators) that influence the delivery of AI tools for chest diagnostic imaging?
	RQ3. What are the experiences of patients and caregivers who had chest x-rays or computed tomography (CT) investigations that were analyzed by staff (supported by AI tools)?	How have patients found the care received as part of the diagnostic pathway (including the use of AI to support diagnostics)? Are patients and caregivers informed or made aware of the use of AI? If so, how? How are results communicated to patients? What are the experiences of patients and caregivers with different demographic and clinical characteristics? Which factors influence patient and caregiver experience of receiving care supported by AI tools in chest diagnostic imaging? (eg, trust and perceptions of AI) What could be done to improve patient and caregiver experiences?
**2**		
	RQ4. What is the impact of using AI for chest diagnostic imaging on service delivery and the wider system?	What is the impact on patients, service delivery, and the wider system? (includes (1) process outcomes such as patient waiting times, processing times, knock-on effects on the overall pathway, ease of use, and resourcing and (2) clinical outcomes such as patient outcomes, safety, and diagnostic accuracy). What levels of AI performance will have a notable impact on these outcomes? What are the implications for patients with different demographic and clinical characteristics, for example, age, sex, and ethnicity? How do data quality and data completeness affect the evaluation of impact? Which factors are likely to be most influential on impact, for example, the function of AI, IT infrastructure, and patient profile? How are implementation approaches and service models likely to influence impact (eg, service delivery and patient outcomes)?
**3**		
	RQ5. What are the cost and resource implications of setting up and delivering AI for chest diagnostic imaging?	What are the implications for staff time, skill mix, and support in implementation and use (including tool maintenance)? How does the implementation of AI for chest diagnostic imaging impact workforce requirements and workload distribution, including changes in staff roles, training needs, and potential shifts in resource allocation across diagnostic and operational workflows? What are the wider resource implications of changes, for example, support at trust and network levels? What are the costs associated with the fee structure—product cost, deployment services, training, and length of license? What are the implications for equity, diversity, and inclusion? Are there any unintended consequences, for example, for the workload?
**4**		
	RQ6. What are the lessons for future implementation and evaluation of AI in diagnostics?	Is AI for chest diagnostic imaging sustainable? Which factors might influence this? How transferable are the lessons to other health care diagnostics settings? How did services and networks use learning from their local evaluation processes? How might local and national evaluation support learning more effectively in the future?

^a^WS: workstream.

^b^RQ: research question.

^c^AI: artificial intelligence.

## Methods

### Design and Theoretical Framework

This is a multisite rapid study that combines qualitative, quantitative, and health economic methods. This evaluation (phase 2) was informed by the findings from the phase 1 evaluation of AI tools for chest diagnostic imaging) [20-22], scoping conversations (eg, with academics, clinicians, policy representatives, professional bodies, and third-sector organizations and regulators) and previous research, and was developed in collaboration with the study Patient and Public Involvement and Engagement (PPIE) group. The evaluation will take place over 12 months (January 2025 to December 2025).

**Figure 1:**
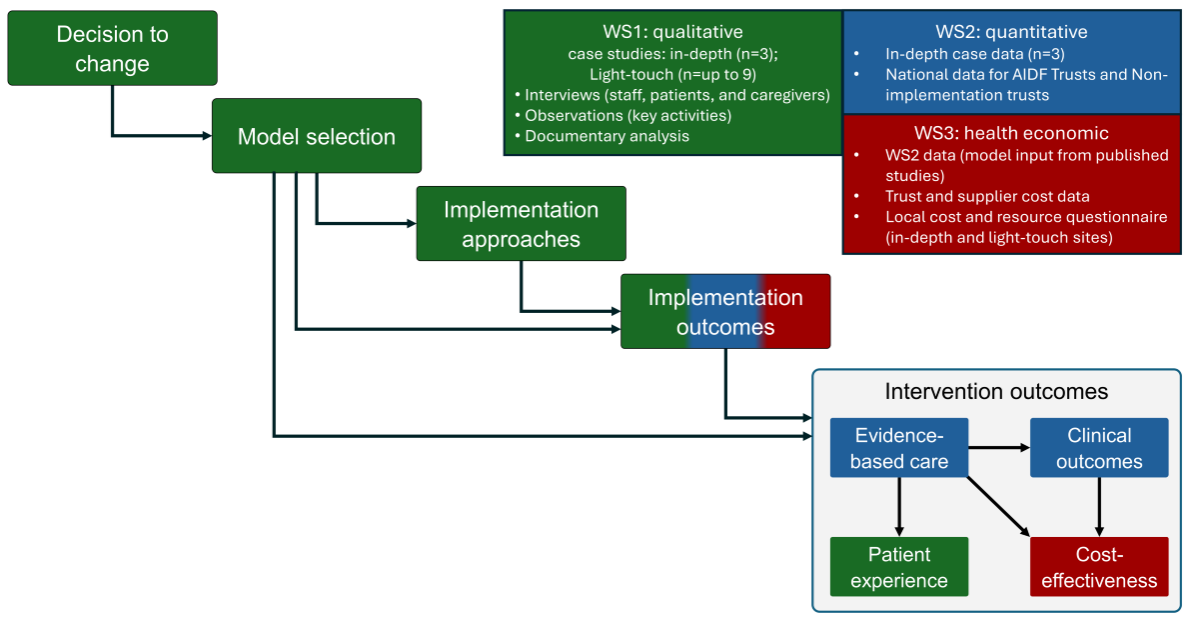
How workstreams will contribute to addressing components of the major system change framework [1]. AIDF: Artificial Intelligence Diagnostic Fund; WS: workstream.

The implementation of AI tools for chest diagnostic imaging may be seen as an example of Major System Change. This study will thus be informed by the Major System Change Framework [26], which was designed to understand the processes, outcomes, and sustainability of such changes, in addition to the relationships between different stages of major system change (Figure 1) [26].

We will employ a 2-level case study design, including 3 in-depth case study trusts and up to 9 light-touch case study trusts, with the aim to ensure our evaluation can contribute both depth and breadth in its lessons across the workstreams (Table 2).

**Table 2:** Summary of activity in in-depth and light-touch case study services.

	In-depth case studies	Light-touch case studies
Number of trusts	3	Up to 9
Workstream 1 activity	Staff interviews (up to 11 per service), with potential follow-up interview of service lead Patient and caregiver interviews (up to 6 per trust) Nonparticipant observations (up to 10 per trust) Documentary analysis	Staff interviews (up to 2 per service), with potential follow-up interview of service lead Documentary analysis
Workstream 2 activity	Local datasets (eg, RIS^a^)	—^b^
Workstream 3 activity	Local datasets (workstream 2) Comprehensive data Cost or resource questionnaire Relevant material raised in workstream 1 interviews	Cost and resource questionnaire

^a^RIS: radiology information system.

^b^Not applicable.

The study has been developed and will be performed in compliance with NIHR RSET equality, diversity, and inclusion requirements, with interpretation of findings and final project write reviewed in consultation with the study PPIE group, advisory group, and wider team (Multimedia Appendix 1).

### Workstreams

#### Workstream 1: Implementation, Staff, Patient, and Caregiver Experience of AI Tools for Chest Diagnostic Imaging (RQs 1-3)

This workstream will focus on the implementation of AI tools for chest diagnostic imaging in NHS services, staff experience with using AI tools for chest diagnostic imaging, patient and caregiver experiences of receiving care supported by AI tools for chest diagnostic imaging, and factors influencing implementation and experiences. The study will be of qualitative design, comprising semistructured interviews, meeting observations, and documentary analysis (Table 3).

**Table 3:** Summary of primary data collection methods within workstreams 1 and 4.

Activity			Study participants		Approx. time
**Workstream 1**					
	In-depth Interviews with staff Interviews with other relevant staff	Up to 32 staff members (10-11 per trust). This will include Staff with direct involvement in AI^a^ for chest diagnostic imaging (eg, radiologists—specialist and general, diagnostic and reporting radiographers, AI suppliers, PACS^b^ and RIS^c^ managers and suppliers, and teams who have been outsourced to provide reporting capacity) Members of the chest or lung MDT^d^ (eg, doctors, nurses, oncologists, and pathologists) Staff with wider oversight or experience of development (eg, information governance teams, clinical safety teams, data managers, project managers, digital and AI leads, and radiology physicists) Imaging network lead Wider system staff (eg, GPs^e^ or ED^f^ staff)		30-60 minutes, with a potential follow-up interview with the service lead toward the end of data collection	
	Light-touch Interviews with staff	Up to 18 staff (up to 2 per trust) Service lead Imaging network lead		30-60 minutes, with a potential follow-up interview with the service lead toward the end of data collection	
	In-depth Interviews with patients and caregivers	Up to 18 patients and caregivers (6 per trust) who have received a CXRg or chest CTh that was supported by the AI tools for chest diagnostic imaging		30-60 minutes	
	Documentary analysis of trust-level documents	Relevant trust level documents pertaining to the implementation of AI for chest diagnostic imaging (eg, project plans, risk documents, meeting minutes, examples of anonymized AI reports, training materials, standard operating procedures, patient pathways, and AI specifications)		N/A^i^	
	Observations of meetings	Up to 30 meetings relevant to implementation of AI tools for chest diagnostic imaging. Meetings include Project meetings Training sessions Trust governance meetings ICBj oversight meetings		Duration of observed meeting	
	Interviews with AIDFk program staff	Up to 3 interviews with the national AIDF program team		30-60 minutes	
**Workstream 4**					
	Online workshops	Two online workshops (n=up to 20 participants) Staff working in services that have implemented AI tools for chest diagnostic imaging (n=1 workshop, 8-10 participants per workshop) Policymakers and other system leaders (n=1 workshop, 8-10 participants per workshop)		60-90 minutes	

^a^AI: artificial intelligence.

^b^PACS: picture archiving and communication systems.

^c^RIS: radiology information system.

^d^MDT: multidisciplinary team.

^e^GPs: general practices.

^f^ED: emergency department.

^g^CXR: chest x-ray.

^h^CT: computed tomography.

^i^NA: not applicable.

^j^ICB: integrated care board.

^k^AIDF: Artificial Intelligence Diagnostic Fund.

#### Sample

### Site Selection

The study sample will comprise 3 of the 66 trusts as in-depth case studies, with up to 9 trusts as light-touch case studies. Site selection will be informed by our learning from the phase 1 evaluation [20-22] and an expression of interest process, whereby all trusts implementing AI for chest diagnostic imaging (through the national meetings) will be contacted for expressions of interest for study participation. Eligibility criteria for site selection are presented in Textbox 1 and for wider data collection in Textboxes 2 and 3. Sites meeting implementation and data quality criteria have been identified through local network engagement and implementation progress tracking, with study inclusion guided in collaboration with imaging network and service leads.

**Eligibility criteria for site selection.**

- All sites will have implemented artificial intelligence (AI) in their chest diagnostic imaging service
- In-depth trusts will have sufficient prospects of good data quality (eg, local picture archiving and communication system [PACS] or radiology information system [RIS] data reports) to facilitate evaluation across workstreams 2 and 3
- Light-touch trusts will not be located in the same networks as the in-depth case studies.
- The study will seek to ensure representation across a range of service characteristics and contexts, including
  The purpose of the AI tool (prioritization, identification of lung cancer vs identification of other chest conditions)
  Scan type (chest x-ray vs chest computed tomography)
  AI tool supplier
  Geographical location (eg, urban, rural, and coastal)
  Other relevant characteristics that will support decision-making (eg, referral pathways, leadership approach [imaging network vs trust], and local PACS and RIS set-up [local arrangement vs regional platform])

**Eligibility criteria for data collection.**

**Staff interview participants**

- The National Health Service staff who work in or with the participating trusts, and who are involved in organization or delivery of care to patients receiving chest diagnostic imaging which have been supported by the artificial intelligence (AI) tools for chest diagnostic imaging; also, Artificial Intelligence Diagnostic Fund (AIDF) program staff
- Over the age of 18 years
- English-speaking or able to participate in an interview with an interpreter
- Able to provide informed consent

**Patients and caregivers**

- Patients and their caregivers (including family members) who have had a chest x-ray or computed tomography (CT) scan that has been supported using AI for chest diagnostic imaging, at one of the 3 trusts included in this study
- Over the age of 18 years
- English-speaking or able to participate in an interview with an interpreter
- Able to provide informed consent

**Workshop participants**

- National stakeholders with relevant job roles (eg, policymakers, commissioners, system leaders, and third-sector organizations) relating to the implementation of AI, or local staff involved in implementing AI from the eleven networks and 60 trusts implementing AI for chest diagnostic imaging as part of the AIDF
- Over the age of 18 years
- English-speaking or able to participate in an interview with an interpreter
- Able to provide informed consent

**Documentary analysis**

- Any documents pertaining to the implementation of AI for chest diagnostic imaging at the participating 3 trusts

**Meeting observations**

- Any meetings relevant to the implementation of AI chest diagnostic imaging at the participating 3 trusts

**Exclusion criteria.**

- Anyone under the age of 18 years
- Anyone who cannot provide informed consent
- Patients and caregivers for which the artificial intelligence tool was not involved in supporting their care
- Patients and caregivers at sites not included in this study

#### Recruitment and Consent: Initial Identification

### Case Study Trusts

To recruit participating trusts, we will present the study plans at existing AIDF network meetings and invite trusts to express interest in taking part. Sampling will be informed by findings from our phase 1 evaluation [20]. In addition, and to ensure a diverse sample, sites will be asked to provide some basic information to enable sites to be purposively sampled (eg, data availability, the purpose of the AI tool, type of scan, supplier, geographical location, referral pathway, leadership approach, and local picture archiving and communication system [PACS] or radiology information system [RIS] setup).

### Staff Interviews

The researchers will work with leads at each site to identify potential staff groups at their trust that may be appropriate for an interview. Researchers will contact potential participants via email to invite them to participate. Staff may also cascade details of the study (and an invite for anyone to contact the researchers if interested) to their staff networks to support recruitment.

### Patient and Caregiver Interviews

The researchers will work with staff leads or R&D contacts at each trust. The staff leads (or research nurses, if available) will contact potential patients and caregivers who meet the eligibility criteria (by telephone, email, or post) to share a study advert and see if they would be interested in participating in the study. Potential participants will be asked to contact the research team directly if they are interested in participating; alternatively, potential interviewees may ask the staff lead or R&D contact to securely pass on their details to the researcher (using the secure UCL Data Safe Haven) if preferred. The researcher will then contact the patient or caregiver to provide further information.

In the first phase of our evaluation, the team learned that services are taking varied approaches to informing patients about the use of AI in the diagnostic process, with some sites choosing not to inform people explicitly. Therefore, the invitation to be interviewed may be the first time patients are made aware that AI supported their diagnostic process: this may cause patients concern or a desire for more information. To accommodate this eventuality, patients will be made aware of the purpose of this study and signposted to national and local sources of information at each stage of the identification and recruitment process, for example, in invitation and recruitment documentation.

The team recognizes that hospital services are extremely busy. Therefore, when recruiting in-depth sites, we will ensure that the proposed approach to identification, invitation, and recruitment is feasible in these sites; further, we will work with local research nurses in sites where they are available to support our work.

Note: For the purposes of patient and caregiver interviews, sites will be classified as Patient Identification Centres.

### Meeting Observations

The researchers will liaise with staff leads at each trust to identify appropriate meetings to observe. For each meeting type, we will liaise with the lead of the event (eg, meeting chair or lead trainer) regarding whether observation will be possible and appropriate.

### Workshops

To recruit workshop participants, we will circulate study adverts via existing AIDF channels and networks, professional groups, social media, local third-sector organizations, and direct invitations.

### AIDF Staff

The researchers will work with the AIDF program leads to identify staff that may be appropriate for an interview. Researchers will contact potential participants via email to invite them to participate.

Details of informed consent processes are presented under the Ethical Considerations section.

#### Data Collection

### Interviews

Interviews will last 30-60 minutes, and will be semistructured and audio-recorded (subject to consent). Interview topic guides have been developed iteratively, informed by phase 1 findings [20-22], scoping conversations, the Major System Change Framework [26], and previous research [32-35].

For in-depth case studies, the aim is to interview up to 32 staff members covering key clinical and organizational roles (Table 3) and sampling patients and caregivers across a range of characteristics, including health outcome following review of scan (and therefore care pathway) and sociodemographic characteristics (eg, gender, age, ethnicity, and disability). We anticipate that patient characteristics may influence how interviewees experience diagnostic imaging services supported by AI (eg, many sociodemographic characteristics may influence AI performance, with underserved groups being disadvantaged). Sampling patients in this way, therefore, ensures a range of perspectives are captured.

The semistructured interviews will be guided by topic guides tailored to stakeholder groups (Multimedia Appendix 2). Interviews will be transcribed verbatim by a professional transcription service and stored securely for analysis by the research team. In light-touch trusts, we will aim to interview up to 18 staff, with members of the NHSE AIDF program team also interviewed for their independent perspective (Table 3).

For all interviewees, sociodemographic information will be sought (on a voluntary basis), including job role and length of time in post (staff) or health outcome, comorbidities, age, gender, ethnicity, disability, sexuality, and employment status (patients and caregivers). All participants will also be informed that they are free to withdraw up to 2 weeks after the date of their interview.

### Staff Interviews

Staff interviews will explore the interviewees’ role and professional background, their views on the reasons and drivers for implementing AI tools for chest diagnostic imaging, the aims, purpose, and function of AI tools, how AI tools are intended to be used and being used in trusts, how care is supported by AI in their trust, their experience of using AI for chest diagnostic imaging (including training, support, etc), perceived impacts and examples of perceived impacts, governance, data monitoring and evaluation, resource use, impacts on (in)equality, unintended consequences of using AI, barriers and facilitators to implementation and delivery, key learnings, and future use. Interviews will be scheduled to take place during regular working hours, as staff are not being compensated for their study participation.

### Patient and Caregiver Interviews

Patient and caregiver interviews will explore the care they experienced, investigations and outcomes received to date (eg, scan received and process of receiving their report), information provision (eg, whether and in which ways they were informed about use of AI), the experience of the care they have received (things they liked and things they disliked), timeliness of care, and barriers and facilitators to their care experience. If previously unaware of the use of AI in their care, they will be provided with a short vignette that explains the use of AI in their local trust followed by questions on their knowledge and views on AI, how it can be used in health care, whether and how the use of AI had been communicated to them, whether they would like to find out more from their care providers (and if so, how), views on the benefits and challenges of AI, perceptions of the impact of AI, possible unintended consequences of its use, and how AI should be used in the future. At the end of the interview, all patients and caregivers will be provided with a secure link to add any further information they would like to share in written format. Participant information sheets and consent forms to be translated, with translation services available for the interview itself, if needed.

### Meeting Observations

Activities to be observed will include up to 30 meetings (up to 10 per in-depth trust) relevant to the use and governance of AI for chest diagnostic imaging, and health care affected by the implementation of AI in chest diagnostic imaging, AI implementation project meetings, AI training sessions, multidisciplinary team meetings, safety and quality committees (directorate- and trust-level), and regional oversight meetings.

### Documentary Analysis

Local documents pertaining to the implementation of AI for chest diagnostic imaging (eg, project plans, risk documents, meeting minutes, examples of anonymized AI reports, training materials, standard operating procedures, patient pathways, AI specifications, local audits, and evaluation plans) will be analyzed across all participating trusts.

#### Data Analysis

A medium Q thematic analysis approach [36], combining inductive thematic analysis and the use of a coding framework [37], will be used to analyze findings. Real-time notes will be used for postinterview completion of Rapid Assessment Procedure (RAP) sheets [38], guided by RQs and the Major System Change Framework [26]. The categories used in the RAP sheet will be based on the interview topic guides. There will be flexibility to add categories during the research process.

Initial themes and subthemes will be developed using inductive thematic analysis [37], the interim findings of which will be shared with key stakeholders throughout the study. The themes developed during the rapid analysis will be applied to interview transcripts and observation field notes to develop an in-depth coding framework for final themes and subthemes with cross-case comparisons made across the case study sites and staff characteristics (eg, to explore barriers or inequities relating to implementation, delivery, and patient experience), where possible.

#### Workstream 2: The Impact of AI Tools for Chest Diagnostic Imaging (RQ 4)

This workstream focuses on evaluating the impact of AI tools for chest diagnostic imaging, performing mathematical modeling analysis on data from sample sites and relevant patient record datasets.

#### Design

Quantitative analysis of data derived from Hospital Episode Statistics (HES), Diagnostic Imaging Database (DID), and NHSE Benefit Register (where available) from all trusts that have implemented AI for chest diagnostic imaging through AIDF (n=63), including all networks involved in the AIDF program. More detailed analysis of imaging data from the 3 in-depth trusts. Given data availability limitations and the immaturity of postimplementation data, mathematical modeling of the chest diagnostic pathway will be informed by the sample data, supplemented with relevant evidence from published studies or the gray literature (these studies need not have investigated the use of AI).

Relevant data collected in workstream 1 will be analyzed to identify factors and implementation approaches likely to influence the impact of AI deployment.

#### Sample

Local data sampled from in-depth sites as described in workstream 1 unless where necessary (eg, due to data extraction or poor data quality issues). All 63 AIDF sites will be included for analyses of data sourced from HES, DID, and NHSE Benefit Metrics where it is available. Trusts that have not implemented AI for chest diagnostic imaging will be included as comparators.

#### Measures

The measures we will investigate will focus on (1) caseloads and workflow (eg, referral volumes, results of tests by category), (2) false positive and false negative results, (3) patient flow and waiting times (eg, time from CXR to CT scan, time to confirmed cancer diagnosis), (4) image processing and reporting (eg, turnaround times), (5) AI use and performance (eg, agreement with clinician, AI failure rates), (6) impact by patient characteristic, (7) influence on early detection (ie, the stage at which cancer is diagnosed), and (8) noncancer pathways outcomes.

False positives will include follow-up CT examinations with negative results (for CXR applications only) and subsequent cancer diagnoses that are negative. False negatives will include cancer cases that are missed by the diagnostic imaging supported by the AI. In all cases, the accuracy of the human reader with the AI tool in a decision support role is being measured.

#### Data Collection

We will seek empirical data from the in-depth sites (aggregated or summary, RIS or PACS system data), together with data from currently accessible resources, for sample and comparator sites: DID, HES, and NHSE Benefits Registers.

A short feasibility study supported by expert advice will also be undertaken to assess the value of HES in its ability to support the analysis of how AI deployment may impact inequalities.

Further data on the performance of AI may be sought from the AI suppliers or trusts, depending on local arrangements, and the availability of linkage to cancer diagnostic data for sites will be explored. The proposed use of each source of data is summarized in Table 4.

The extent to which we can investigate these metrics will depend on what we are able to glean from these data sources, and their quality and completeness. Since many sites are in the early stages of deployment, this is not yet clear, and the influence of data quality and completeness is included within the workstream.

For example, clinical outcome measures can only be obtained from sites where data are linked between radiology systems and cancer registries, allowing us to chart patients’ diagnostic journeys to a definitive clinical outcome.

Understanding how outcomes differ for different types of patients, that is, understanding implications of deployment on inequalities, will require access to record-level data available from HES. The value of HES in supporting this analysis will need to be explored first to understand its capabilities and limitations, so, to this end, we will undertake a short feasibility study supported by expert advice.

Where we plan to use DID or HES for comparators, we will use longitudinal data both before and after deployments and apply statistical methods that will account for deployment at different times. When using patient-level data, trust factors can be included as random effects, and we could simultaneously explore any influence of patient characteristics. We cannot, however, account for simultaneous interventions that may be happening in comparator sites that aim to improve backlogs.

We will work alongside workstream 3 to develop a model of the lung cancer patient pathway supported by site data alongside available evidence from published sources on diagnostic accuracy, including resource constraints and efficiency. These will map the progress of patients from initial tests through to any confirmed cancer diagnosis with progressions dependent on the underlying cancer stage. The purpose of these models will be to link different levels of AI performance to outcomes such as volumes of follow-up tests, missed cancer diagnoses, and stage of cancer at first diagnosis. This would lead to more generalizable findings.

Once we scope data in HES and DID, we will know the degree to which we can investigate process outcomes (imaging processing, patient flow, etc). After speaking with individual sites regarding their data linkage, we will know whether we can look at clinical outcomes and match radiology data with the cancer registry. We will use existing evidence as well, but acquiring local data is important in centering this analysis around AIDF sites.

The nature of any potential bias that might affect our quantitative results have been, or will be, discussed with advisors, other stakeholders, and sites. We have attempted to request data from local sites and national datasets that include factors related to potential bias, where available. These will then be adjusted for in our analyses. Mindful that each trust organizes its local data differently, we are being thorough in our understanding of each field of data (in the form of continued correspondence with local data managers) to ensure that bias resulting from incorrect interpretation does not occur.

We will ensure that we define metrics that are less likely to be affected by missing data, that is, where a sample of cases will provide sufficient information. If there is missing data from local sites and it is important to our analysis, we will liaise with those sites to see if the issue can be rectified. In some cases, we can use proxy measures, for example, numbers of suspected cancers after x-ray as a proxy for numbers of follow-up CT scans.

Together with the work performed in workstream 3, these data and analyses will be used to develop a model of the lung cancer patient pathway from initial referral through to any confirmed cancer diagnosis. Evaluation of AI deployment will thus be enabled via generalizable study findings from the linkage of the impacts of AI deployment to diagnostic imaging and care pathway outcomes (eg, follow-up test volumes, missed cancer diagnoses, and stage of cancer at first diagnosis).

**Table 4:** Proposed data sources for workstreams 2 and 3.

Data source	Proposed use	Advantages	Disadvantages
NHSE^a^ Benefits Registers	Measures relating to case volumes, the change in processing times, times to follow-up tests, resourcing, and cancer diagnoses Measured at baseline and postdeployment (6 months, 12 months) Used for metrics not covered by other sources Mainly descriptive analysis	Available for all sites Data submission requirements of each site in order to obtain AIDF^b^ funding Ranges across most of the relevant outcomes	Historic baseline available only Processing times measured as averages Not all data can be provided by all sites Incomplete recording of new data fields (eg, image prioritization categorization) Lacks the granularity required for study aims
Data from local sites	Data underpinning the Benefits Register metrics available in greater detail Within site exploration of variation by patient characteristics Within site comparisons, for example, patients with normal and abnormal imaging results Exploration of issues arising from workstream 1 interviews Findings from local studies	Can request greater data granularity Can obtain greater detail on processing times Can analyze data by patient characteristics Potential to analyze specific noncancerous conditions (eg, infections, pulmonary embolism)	Since these data inform the Benefits Registers, the issues of data completeness are the same Agreements need to be put in place with each site we select Aggregated data may restrict levels of granularity due to suppression of low numbers
DID^c^	Analysis of outcomes relating to processing times. Comparison with non-AIDF sites	Can be obtained for all sites and non-AIDF sites for comparison Data completeness and quality assessments are also issued alongside publication Longitudinal data Can obtain greater detail on processing times Can analyze data by patient characteristics	Only available for a limited number of outcome measures Approximate 5-month time lag Aggregated data may restrict levels of granularity due to suppression of low numbers Cannot identify patient cohorts with abnormal chest x-ray results
HES^d^	This depends on the outcome of the feasibility study. Potential outcomes include times between diagnostic tests and treatments. Analysis of differences due to patient characteristics	Easily available Patient-level longitudinal data Can analyze data by patient characteristics Can analyze data for non-AIDF sites for comparison Can isolate GP^e^ referrals	Feasibility is uncertain. It may lack some required detail Diagnostic information in outpatient records may be limited, particularly because the type of procedure performed is not always specified in the data Unable to distinguish suspected lung cancers from other reasons for CT^f^ referral Approximate 3-month time lag
Postmarket surveillance data	Assessing the performance of the AI^g^ tools such as clinician-tool agreement and AI failure rates	Probably the only source of these data	Agreements may need to be put in place with the suppliers
AI supplier cost data	Key cost component for evaluation of AI deployment in workstream 3	Cost data collated for all suppliers involved with the procurement process across all trusts in the AI deployment	Aggregate estimates will not be fully representative of the specific costs applicable to individual trusts
Published AI platform performance metrics (sensitivity, specificity)	False positive and false negative result rate estimates (where non-AI diagnostic imaging or AI supplier data are unavailable)	Published large sample study data	Published data may not reflect real-world service performance, given the variation in care pathways and AI platform application
Participant trust questionnaires	Collection of resource use and cost data relevant to the AI deployment and patient diagnostic pathway (eg, staff type, numbers, and time, equipment, and IT infrastructure)	Able to pose questions specific to the requirements of workstream 3	Knowledge required for completing the questionnaire may not reside with one individual or group Risk of inaccuracy, bias, and generalizability for retrospective anecdotal evidence
Published lung cancer patient pathway cost and outcome data^h^	Will be used to populate the care pathway are beyond the scope or resource capacity of the RSETi project	Peer-reviewed estimates of costs and health outcomes for lung cancer patients in the UK population	The need to assume that the published data are generalizable to the patient sample in the participating trusts

^a^NHSE: National Health Service England.

^b^AIDF: Artificial Intelligence Diagnostic Fund.

^c^DID: Diagnostic Imaging Database.

^d^HES: Hospital Episode Statistics.

^e^GP: general practice.

^f^CT: computed tomography.

^g^AI: artificial intelligence.

^h^By stage of diagnosis or associated with false negative or false positive diagnosis.

^i^RSET: Rapid Service Evaluation Team.

#### Workstream 3: The Cost and Cost-Effectiveness of AI Tools for Chest Diagnostic Imaging (RQ 5)

This workstream focuses on evaluating aggregate costs and cost-effectiveness of AI tool deployment in the diagnostic chest imaging stage of the lung cancer care pathway, across a small sample of participating sites for chest diagnostic imaging.

#### Design

**Figure 2:**
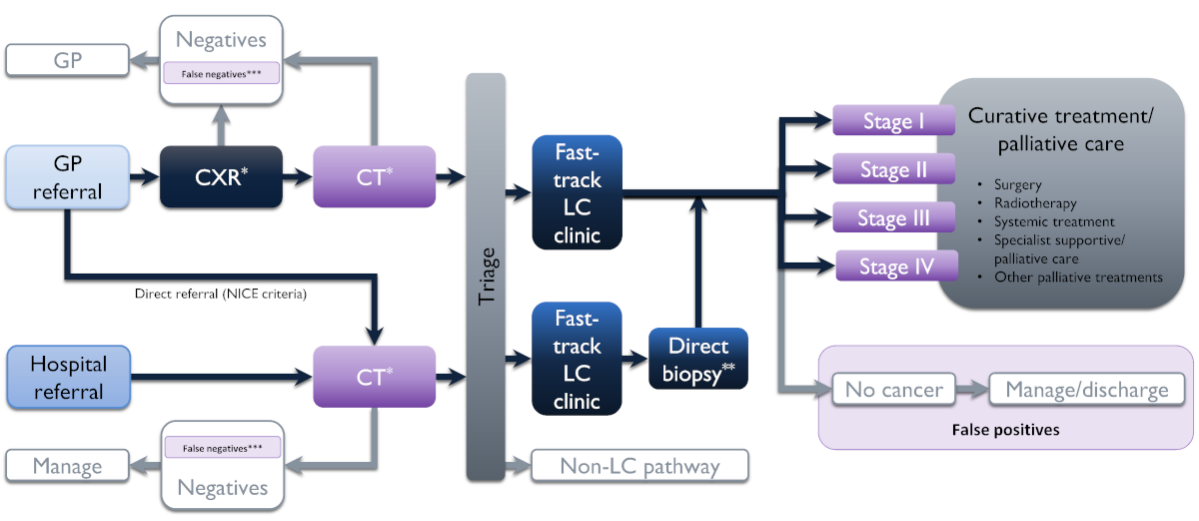
Overview of the lung cancer diagnostic pathway. Adapted from National Optimal Lung Cancer Pathway, NHS England, 2020 [40]. A&E: accident and emergency; CT: computed tomography; CXR: chest x-ray; GP: general practice; LC: lung cancer; NICE: National Institute for Health and Care Excellence. *Includes follow-up CT or CXR for patients with indeterminate results. **Rapid diagnosis pathway, where detailed staging and fitness investigations are not needed to guide management (eg, patients with advanced disease not suitable for curative intent treatment). ***False negatives are presumed to re-present at A&E.

Given the limitations of the resource capacity and timeframe of this project, a pragmatically designed economic model of the lung cancer care pathway will be developed, mapped to a simplified version of the National Optimal Lung Cancer Pathway (Figure 2) [39].

The model will adopt a decision tree approach with an NHS and Personal Social Services perspective, and a lifetime time horizon. Patient flow and short-term costs and outcomes from the chest diagnostic imaging stage of the care pathway will be informed by data collected in this study. Long-term costs and outcomes for lung cancer patients (by stage at diagnosis) will be derived from published estimates from relevant studies in a UK setting and from appropriate health service datasets.

The comparator for AI deployment will be the usual care pathways for chest diagnostic imaging predeployment (without AI assistance). Data for these pathways will be drawn from the same sites pre-AIDF implementation, plus data from sites where AI tools for chest diagnostic imaging have not been implemented.

#### Sample

The model will be populated by relevant data collated and synthesized from workstream 2 (local datasets from the in-depth participant trusts), responses to a participant questionnaire (completed by both in-depth and light-touch participant trusts), relevant AIDF material (eg, procurement documentation), and any relevant information raised during participant interviews in workstream 1. Data required for populating the model, which is otherwise unavailable from these sources (eg, beyond the time or resource scope of this project), will be obtained from relevant health care datasets or published studies.

Where comparative (AI vs non-AI) estimates are used to inform model input parameters, these will be evaluated relative to baseline data from the respective sites, or from sites which do not use AI in the care pathway (where available). Pre- and postdeployment data obtained during the collection period of the evaluation will be converted to annualized estimates for costs and outcomes, weighted by the respective chest diagnostic imaging activity of the participating trusts.

#### Measures

Relevant measures for informing the economic model are outlined in Table 5. Those to be obtained (where possible) from this study will include the measures listed in Textbox 4.

Measures for the economic modeling obtained from published studies or appropriate published datasets will include the items listed in Textbox 5.

**Table 5:** Key inputs for the economic model^a^.

Description			Category							Source^b^
			Care pathway		Cost		Outcome			
**GP^c^ referrals**										
	Total referrals, CXR^d^ and CT^e^ (n)	✓		✓				A		
	Referrals to CXR (n or % of total)	✓		✓				A		
	Referrals to CT scan (n or % of total)	✓		✓				A		
	Positive predictive value, CXR (%)	✓						A, B		
	Positive predictive value, CT (%)	✓						A, B		
**Hospital referrals**										
	Total referrals, CT (n)	✓		✓				A		
	Positive predictive value, CXR (%)	✓						A, B		
	Positive predictive value, CT (%)	✓						A, B		
**Chest imaging**										
	CXR costs (AI^f^, non-AI)	✓		✓				A, C		
	CT costs (AI, non-AI)	✓		✓				A, C		
	CXR sensitivity and specificity (AI, non-AI)	✓		✓				A, C		
	CT sensitivity and specificity (AI, non-AI)	✓		✓				A, C		
	AI imaging failure rate (CXR, CT)			✓				A		
**Post imaging**										
	Confirmatory diagnostic testing, true positive	✓		✓		Disutility		A, B		
	Confirmatory diagnostic testing, false positive	✓		✓		Disutility		A, B		
**Fast-track LC^g^ clinic**										
	Clinic MDT^h^, no biopsy (n or % of total)	✓		✓				B		
	Clinic MDT, biopsy (n or % of total)	✓		✓		Disutility		B		
**Curative treatment or palliative care**										
	Treatment or care, by stage of diagnosis, true positive (n or % distribution)			✓		LY^i^, QALY^j^		B		
**False negatives**										
	Patient re-presentation (GP, A&E^k^)	✓		✓		Disutility		B		
	Chest imaging, confirmatory diagnostic testing, fast-track clinic (±biopsy)	✓		✓		Disutility		A, B		
	Treatment or care, by stage of diagnosis (n or % distribution)	✓		✓		LY, QALY		B		
**Other metrics: clinical safety**										
	AI incidents (Datix reports)	—^l^		—		—		A		

^a^Inputs applicable for care pathways with the use of AI in either CXR or CT scans.

^b^A: RSET study data; B: health care datasets or published studies; C: supplier data.

^c^GP: general practice.

^d^CXR: chest x-ray.

^e^CT: computed tomography.

^f^AI: artificial intelligence.

^g^LC: lung cancer.

^h^MDT: multidisciplinary team.

^i^LY: life years.

^j^QALY: quality-adjusted life years.

^k^A&E: accident and emergency.

^l^Not applicable.

**Measures collected for economic modeling.**

- Patient caseloads and workflow (eg, referral volumes and results of tests by category)
- False positive results (based upon sites' data or published specificity data for non–artificial intelligence (AI) diagnostic imaging and from AI suppliers, where the former are unavailable)
  Costs and outcomes subsequent to false positive results will be modeled on the assumption that patients progress for diagnostic testing and multidisciplinary team confirmation of no lung cancer, prior to discharge
- False negative results (based upon sites' data or published sensitivity data for non-AI diagnostic imaging and from AI suppliers, where the former are unavailable)
  Costs and outcomes subsequent to false negative results will be modeled on the basis that patients present to either their general practice or accident and emergency (A&E) and are referred for diagnostic imaging following a delay after receipt of the false negative result, with a corresponding progression in stage of lung cancer at diagnosis
- Any costs and outcomes associated with patient flow and waiting times (eg, training for rapid delivery of results to patients, time from chest x-ray to computed tomography (CT) scan, time to confirmed cancer diagnosis)

**Measures from published studies or datasets.**

- Distributions of cancer diagnosis, by stage (after true positive or false negative results)
- Post—chest imaging costs for confirmation of diagnosis (eg, multidisciplinary team case review, follow-up diagnostic and staging tests, and biopsy)
- Costs and health outcomes associated with lung cancer by stage of diagnosis, or with a false positive or false negative result

Costs and outcomes associated with other (noncancer) conditions which may diagnosed via chest imaging (incidentally or by specific request) will be out of scope for this evaluation.

#### Data Collection

Further to data relevant to the economic evaluation, which will be collected in workstream 2, data will be sought from the following sources.

Site-specific data on deployment, and AI use and performance (eg, staff type or mix, image assessment and reporting time, AI failure rates), will be sought via a data collection questionnaire which will be distributed by the project team to trusts during the project data collection stage.

AI supplier cost data are available in a protected area of the FutureNHS web portal. Owing to its commercial sensitivity, these data will be aggregated prior to use or publication.

#### Data Analysis

The proposed use of each source of data is shown in Table 4. To address concerns about the comparability of sites, we will use statistical adjustment for trust-specific and patient-specific characteristics (eg, age, comorbidities, and socioeconomic status) to control for differences between sites and populations. This approach will ensure that any observed differences in outcomes between AI and non-AI sites are not confounded by these variables.

Alternative models of AI implementation (eg, case prioritization, decision support, and autonomous AI reporting of high-confidence normal cases) will be explored for their relative potential impact on organizational and clinical outcomes.

#### Workstream 4: Developing Lessons to Inform Future Implementation and Evaluation of AI for Chest Diagnostic Imaging

This workstream will integrate findings from workstreams 1 to 3 and develop lessons to inform future implementation programs and evaluations (RQ 6, Table 1; see Table 4 for primary data collection methods).

#### Design

Integration of the workstreams will take place throughout the evaluation to enable complementarity of the workstreams (eg, workstream 1 has ensured interview topic guides cover issues relevant to workstreams 2 and 3; workstreams 2 and 3 will seek to analyze quantitative and resource data that are relevant to themes emerging from workstream 1; and workstreams 2 and 3 will collaborate on modeling work). Findings from workstreams 1 to 3 will be synthesized and then triangulated across workstreams (eg, aligning relevant workstream 1 findings on perceptions of impact, resource use, and explanatory factors to workstreams 2 and 3). Further, weekly meetings will enable qualitative, quantitative, and health economic researchers to share findings and interpretations across workstreams. Findings and developing recommendations will be integrated around our RQs and the Major System Change framework and will be presented at 2 stakeholder workshops.

#### Stakeholder Workshops

We will hold 2 online workshops with up to 20 participants (8-10 per workshop). The details of participants and workshop format are presented in Table 6. Further to the workshops, stakeholders will also be invited to provide further feedback during the development of drafts relating to specific findings (eg, quantitative evaluation guide).

**Table 6:** Stakeholder workshops.

Workshop	Participants	Workshop format
1: Staff	8-10 participants, including Networks and trusts that are implementing AI^a^ AIDF^b^ and non-AIDF sites	RSET^c^ present Findings and lessons learned (workstreams 1-3) Recommendations on Implementation of AI for chest diagnostics Future research and evaluations
2: National stakeholders	8-10 participants, including Payers Policymakers System leaders Third-sector organizations	Participants discuss and make recommendations on Study findings and lessons Future and sustainability of AI tools Chest diagnostics Radiology diagnostics more generally

^a^AI: artificial intelligence.

^b^AIDF: Artificial Intelligence Diagnostic Fund.

^c^RSET: Rapid Service Evaluation Team.

#### Data Collection

Information sheets will be sent to interested participants, and consent for data collection will be sought in advance of the online workshops. Each workshop will last between 90 and 120 minutes, with discussions audio-recorded on an encrypted Dictaphone (subject to consent) and detailed notes taken to capture key findings. Recordings will be transcribed verbatim by a professional transcription service, anonymized and kept in compliance with General Data Protection Regulation (GDPR) 2018 and Data Protection Act (2018). Participants will be informed that while they can withdraw from the discussion, any data provided up until that point will be kept as it would not be possible to remove individual data from group discussions.

#### Data Analysis

Researchers will analyze workshop findings using inductive thematic analysis [37], organized around key themes and findings from workstreams 1 to 3. Workshop findings will support the validation and further development of recommendations for AI implementation and evaluation that result from this work.

#### Ethical Considerations

##### Human Subject Ethics Review Approvals

Based on the Health Research Authority (HRA) decision tool and consultation with the UCL and UCLH Joint Research Office, most components of this evaluation (staff-focused qualitative work, quantitative work, and health economic work) can be classified as a service evaluation; these evaluation components have received ethical approval from the UCL Life and Medical Sciences Research Ethics Committee (Project ID 0603). The patient and caregiver-focused qualitative work has received HRA ethical approval from the North West – Greater Manchester West Research Ethics Committee (Project ID 25/NW/0137).

Although this is a relatively low-risk evaluation, we are aware of the sensitive nature of this work for organizations and individuals, especially patients and caregivers. The research team has experience in conducting health and care research on similarly sensitive topics. We will maintain the independence of the research, follow an informed consent process, and maintain the anonymity of participants and organizations.

##### Informed Consent

###### Interviews and Workshops

All potential interviewees upon expressing interest in participating will be sent a participant information sheet and consent form (either by email or post, depending on preference). They will be given at least 48 hours to review the information and consider whether to participate. Participation is fully voluntary, and this is communicated clearly on recruitment documentation. If potential interviewees are happy to take part, they will be asked to provide consent prior to the interview or workshop. An informed consent process using participant information sheets and written consent (scanned forms or typewritten or electronic signature), or audio-recorded verbal consent will be used for recruitment to ensure and demonstrate informed and voluntary participation. If participants would prefer to post consent forms back, they will be sent a prepaid envelope to support this. If patients are not able or willing to take part in the interview but would still like their views to be included, we will ask patients if we can approach their caregiver (if they have one) to capture their perceptions of the patient’s journey and overall experience with the service.

For interviews, the researcher will then arrange a time to conduct the interview over the phone or an online platform (Zoom or Microsoft Teams). If preferred or an individual interview is not possible, staff can choose to take part in a joint or group interview (where feasible). Similarly, patients and caregivers can choose whether they would like to take part in an interview separately or jointly.

For the workshop, participants will be sent the link to join once consent has been provided. Participation is fully voluntary, and this is communicated clearly on recruitment documentation.

##### Meeting Observations

We will send the chair or event lead the information sheet and consent form. Participation is fully voluntary, and this is communicated clearly on recruitment documentation. If the event lead is happy in principle for the meeting to be observed, they will be asked to provide written consent, and information and consent forms will be shared with event attendees for information. Researchers will offer to present to the meeting an overview of the evaluation and what observations will involve. At the start of each meeting, we will also gain verbal consent from meeting or event attendees for the study team to observe and take anonymized notes.

#### Privacy and Confidentiality

##### Qualitative Data (Workstreams 1 and 4)

Participant interviews and workshops (qualitative data) will be recorded on an encrypted, password-protected digital recorder (only the researcher will know the password). Data will be collected by a team of qualitative researchers from RSET. Staff, patient, and caregiver interview, observation, and workshop consent forms, audio recordings, anonymized notes, and any documents received will be securely transferred using the Data Transfer portal onto the UCL Data Safe Haven (DSH, a secure electronic environment, certified to ISO27001 information security standard and conforms to the NHS Information Governance Toolkit). Once transferred onto the UCL DSH, the data will be cleared from the Dictaphone.

Any participant consent forms received via post will be sent to our RSET team members at University College London (UCL) and securely transferred onto the UCL DSH. Paper copies will be securely destroyed once scanned and uploaded to the UCL DSH. Electronic copies of consent forms received via email will be transferred onto the UCL DSH. If patients and caregivers would prefer to provide written consent or submit responses to sociodemographic questions using a secure survey link instead of verbally during the interview, the research team will develop online survey versions using the platform REDCap (Research Electronic Data Capture; Vanderbilt University). Patients and caregivers would be sent a link, and responses would be returned directly into the UCL Data Safe Haven via REDcap and would only be accessible by the qualitative research team.

Digital audio recordings of participant interviews and workshops will be sent to a UCL-approved contractor for transcription (TP Transcription Limited). Transcripts will be fully anonymized (names and places) and organized by participant codes. Anonymized transcripts and other relevant data will be stored in a secure folder to which only the named researchers (RSET qualitative team) have access. Only the research team will have access to participants’ personal data (ie, name and contact details). Participant identifier codes will be stored in the UCL DSH and kept separate from study data.

##### Impact and Cost Data (Workstreams 2 and 3)

Trust and network-level benefits registers will be accessed via NHSE and transferred to the UCL DSH, where it can only be accessed by members of the research team. These data are aggregated across the deployment sites and will, therefore, not contain any person-identifiable information.

Lung cancer diagnostics and outcome data from individual sites will not contain any person-identifiable information and will be processed within the UCL DSH (where it can only be accessed by members of the research team), after transfer from the network sites via the FutureNHS website**.** These data will either be aggregated with low numbers suppressed or in the form of summary statistics reflecting data distributions (eg, medians and standard errors of turnaround times). Data sharing agreements will be drawn up for each site from which we request data.

Data from the DID will come from NHSE and in an aggregated form. This will be kept within the AI folder on the Nuffield OneDrive for analysis. An appropriate data acquisition form will be completed.

HES outpatient, inpatient, and emergency care datasets are held by the Nuffield Trust and stored on its secure server. An agreement is already in place with NHSE to allow Nuffield Trust staff to use these data for NIHR RSET projects. Staff undertaking patient interviews will have no access to these data and, similarly, all details on patient interviews will be stored on the UCL DSH in an area that will be inaccessible to staff using the HES datasets. This mitigates against interviewed patients being identified in HES.

AI supplier cost data will be held on DSH and will be accessed from a protected area of the FutureNHS. Data will be anonymized and aggregated to mean overall estimates for the respective diagnostic imaging method, prior to use. Returned participant questionnaires will be securely transferred onto the UCL DSH (electronic copies), and if applicable, paper copies will be securely destroyed once scanned and uploaded to the UCL DSH.

Our use of HES is covered by an agreement with NHSE for its use in RSET projects, which will cover secondary analysis without consent (ref: DARS-NIC-194629-S4F9X). Other national data (DID) is aggregated, so no such approval is necessary. Data received from trusts is either nonpersonal aggregated data or, if patient-level, received under terms agreed by the trust that covers the use of the data for secondary analyses without primary consent.

##### Data Handling and Management (All Workstreams)

A Data Sharing and Processing Agreement is in place between the research team and NHSE for the purposes and duration of this evaluation. This covers documents held and developed by the AIDF program.

The study is compliant with the requirements of GDPR (2016/679) and the Data Protection Act (2018). All researchers and study site staff will comply with the requirements of the GDPR (2016/679) with regard to the collection, storage, processing, and disclosure of personal information, and will uphold the Act’s core principles.

UCL will act as data controller for this study, with principal researcher (AIGR) leading on associated processes. He will process, store, and dispose of all data in accordance with all applicable legal and regulatory requirements, including GDPR and the Data Protection Act (2018) and any amendments thereto. Only relevant and necessary data will be collected in line with the aims of this study. Data will not be transferred to any party not identified in this protocol and are not to be processed and transferred other than in accordance with the participants’ consent.

In line with GDPR guidelines on data minimization, we are only collecting personal data that is relevant and necessary for this study.

#### Compensation Details

Participants will receive no compensation or incentive to take part in this evaluation.

##### PPIE

Members of the PPIE group have and will continue to contribute extensively throughout the life of the project. These contributions will include participation in dedicated PPIE workshops, attendance of study team meetings, and supporting the development of study methodology, the consideration of key findings, and the drafting of public-facing outputs for dissemination (Multimedia Appendix 1).

## Results

As of October 2025, trust-level research and development approvals with participating sites are complete, and data collection has commenced. Results are expected to be reported by the end of February 2026.

## Discussion

### Expected Findings

This protocol describes the methodology for a mixed methods study aimed at providing a greater understanding of the use of AI tools in chest diagnostic imaging.

The study comprises four concurrent workstreams: (1) qualitative exploration of the facilitators and barriers to the implementation of AI in chest diagnostic services, and views on AI implementation from patient, caregiver and health service member perspectives; (2) quantitative evaluation of the impact of AI tools on chest diagnostic imaging, based upon mathematical modeling analysis of key service metrics data from sample sites and from relevant patient record datasets; (3) evaluation of aggregate costs and cost-effectiveness of AI tool deployment in the diagnostic chest imaging stage of the lung cancer care pathway; and (4) integration of findings from workstreams 1 to 3 for the development of recommendations to inform future implementation programs and implementation evaluations.

This study builds on the work in the phase 1 study [24], which reinforced the appreciation of a sparse landscape for publications on the real-world use of AI, including the perspectives of patients, caregivers, or general public, or the effects of AI on inequalities and service costs. Despite the potential for AI to help with diagnosis and an initial overall positive outlook from health care staff on the future use of AI, implementation for chest diagnostic services took longer than anticipated. The process of implementation was resource-intensive, covering multiple tasks including AI tool procurement, ensuring it worked safely within hospital systems, meeting clinical and organizational governance requirements, and establishing service audit and performance monitoring frameworks. Initial indications of facilitators (eg, support/input from specialists or experienced colleagues) and barriers (eg, dedicated time and resources) to the implementation process were also identified.

These findings provide a framework from which the phase 2 study can explore these and other areas of interest in greater breadth and depth. This will result in the production of a complementary series of findings which will inform best practice for communicating the role and benefits of AI in health care, the advancement of AI adoption in health care service delivery, and in the provision of robust evidence for future health service planning.

### Limitations

We acknowledge several limitations in this study, some inherent to rapid evaluations, and others specific to the context of AI deployment.

It is noted that participating sites are “early adopters” of AI technology in the context of chest diagnostic imaging: this may represent a risk in the generalizability of lessons identified. To help address this, we will seek to include later adopting services within our light-touch sample. We will also explore the extent to which our sampling represents a limitation to the learning generated in any resulting reports from this evaluation.

Our qualitative workstream will collect data from most of the imaging networks participating in AIDF and capture evidence from services that use AI tools differently in diverse organizational contexts. However, it is likely that we will be unable to analyze the full range of experiences of implementing AI tools. Our light-touch sites will capture less data and from fewer stakeholders and will not include patient and caregiver perspectives. Our patient and caregiver sample will cover a diverse range of backgrounds and experiences but will be unable to capture the full range of different patient groups experiencing AI-supported diagnostics. Finally, we will be unable to observe clinicians reading AI-supported diagnostic outputs.

Although national datasets will be used for analysis where applicable, participating sites for the study will be limited to a small sample with sufficiently mature postimplementation data for analysis and that are able to ensure timely access to their data. While we seek to be able to evaluate whether observed changes in these sites are significant, generalizable findings from their data alone may be difficult or potentially not possible. Moreover, the expected accessibility of data, timescales for project and site implementation, and the linkage of data across the care pathway will affect the extent to which we can explore and draw inferences from key service metrics, may preclude the observation of longer-term impacts (eg, stage of cancer at diagnosis) and the ability to chart patient journeys through the care pathway.

The AIDF program was implemented at a time when many networks and trusts were simultaneously experiencing interventions aimed at addressing service backlogs as a consequence of the COVID-19 pandemic. In our analysis, it will not be possible to account for the impact of these simultaneous interventions in the comparator sites.

For the economic modeling, we will endeavor to ensure that population of model parameters for the comparator (usual care) pathway is as robust as possible. We acknowledge, however, that this will depend on the availability and quality of data from the relevant sources, and where these are insufficient, appropriate data from existing studies will be sought to inform the model.

### Conclusions

AI has the potential to transform the delivery of health care across multiple clinical specialisms, in terms of improvements to diagnostic accuracy, error reduction or prevention, productivity, clinical decision-making, and reducing workforce burden. To date, however, there is mixed evidence for the realization of this potential, which is a risk given the notable financial and resource investment that would likely be required for a national implementation program.

Obtaining greater insights into facilitators and barriers to the adoption of AI technology, perceptions of both the general public and health care staff on the roles AI may play in health care delivery, best practice for the implementation of AI into existing care pathways and service infrastructure, accurate service performance evaluation, and the development of models to best support evidence-based decision-making, are important in establishing a framework upon which the greatest benefits of the use of AI in health care can be realized.

## Appendix

Multimedia Appendix 1 Additional information on governance and oversight.

Multimedia Appendix 2 Topic guides for semistructured interviews.

## Abbreviations

AI: artificial intelligence
AIDF: Artificial Intelligence Diagnostic Fund
CT: computed tomography
CXR: chest x-ray
DID: Diagnostic Imaging Database
GDPR: General Data Protection Regulation
HES: Hospital Episode Statistics
HRA: Health Research Authority
NHS: National Health Service
NHSE: NHS England
NIHR: National Institute for Health and Care Research
PACS: picture archiving and communication system
PPIE: Patient and Public Involvement and Engagement
RAP: Rapid Assessment Procedure
REDCap: Research Electronic Data Capture
RIS: radiology information system
RQ: research question
RSET: Rapid Service Evaluation Team
UCL: University College London
UCL DSH: UCL Data Safe Haven

## Data Availability

The availability of original data for the study will be restricted subject to data sharing agreements for the participating trusts and for electronic patient record dataset access, in accordance with their terms of use (eg, Hospital Episode Statistics).
