# Supplementary material for: Implementation, Experiences, Impact, and Costs of Artificial Intelligence in Chest Diagnostics: Protocol for a Mixed Methods Evaluation
Source: JMIR Res Protoc. 2025 Oct 31;14:e81421. doi: 10.2196/81421 (PMC12619010; doi:10.2196/81421)
Supplement: Multimedia Appendix 1 [file resprot_v14i1e81421_app1.docx]

# Additional Information on Governance and Oversight

## Peer and Regulatory Review

### Peer review

This study protocol has been peer reviewed in accordance with UCL/UCLH requirements. It was peer reviewed by three reviewers external to UCL, with a diverse range of relevant clinical and academic expertise. It was also reviewed by the NIHR.

### Governance

This project is led by AIGR and delivered by a team of researchers and patient and public representatives. The research team meets on a weekly basis, with a set agenda that includes updates on progress of the AIDF programme, workstream-specific updates, project timeline, risk management, opportunities for dissemination and impact, and dedicated sections on PPIE and EDI (See sections B and C for details). In addition, the project lead will report on progress to the RSET Executive Management Group monthly meetings, with a focus on progress, quality assurance, troubleshooting, and emerging learning and potential implications.

Independent oversight and advice will be provided in the following ways. Our project is supported by two independent clinical advisors. Secondly, a dedicated Evaluation Advisory Group, featuring independent stakeholders (including clinical, academic, and patient and public perspectives) will meet approximately three times at key stages of the study. Second, the study will be discussed at the RSET Stakeholder Advisory Board, which includes a range of clinician, academic, PPIE, and EDI experts, and meets every 6 months to offer oversight, challenge, and advice. Finally, we will update the AIDF evaluation subgroup regarding project progress and findings on a regular basis.

## Patient and Public Involvement and Engagement

Patients and the public have been and will continue to be central to this study. Our team includes the RSET PPIE co-lead and two public contributors with an interest in chest diagnostic imaging. These contributors attend project meetings and have actively shaped the development of this protocol.

Specific changes made to the protocol as a result of PPIE input include:

Refining the research questions to better reflect patient concerns around trust, communication, and diagnostic timelines. We also conducted a dedicated PPIE workshop with four members of the public with relevant lived experience. Their feedback supported our proposed design and helped refine recruitment materials and methods for explaining AI in accessible terms.

Looking ahead, public contributors will be actively involved in:

Interpreting qualitative findings, including coding workshop participation co-authoring study outputs, including academic articles, lay summaries, and policy briefs supporting dissemination through presentations, public-facing materials, and stakeholder engagement activities. Additionally, we will continue to engage with national public-facing organisations (e.g., Cancer Research UK, National Voices, Understanding Patient Data) to ensure broader perspectives are included. Throughout the study, we will regularly check in with our public contributors to gather feedback on their experience and ensure their contributions are meaningfully supported.

All involvement will be supported by the RSET PPIE leads and project manager, and contributors will be compensated in line with NIHR INVOLVE guidance.

## Equality, Diversity and Inclusion (EDI)

To ensure that our project thoroughly and comprehensively considers issues of equality, diversity and inclusion, we will review compliance with our NIHR Rapid Service Evaluation Team (RSET) EDI assessment tool (see Table S2) at two stages during this project: (i) during development of the project evaluation and (ii) following data collection and analysis. The tool covers EDI considerations throughout the whole project, including when building the initial team, drawing on published EDI frameworks to consider EDI aspects relevant to the evaluation during the discovery and scoping phases, protocol development, stakeholder engagement, data collection, data analysis, and dissemination.

To date, the team have explored EDI considerations with our PPIE panel and with relevant stakeholders during scoping discussions, built EDI considerations into RQ development, and have considered issues of EDI when developing this protocol (for example when considering site and participant selection).

## Table S1. Equality, diversity, and inclusion (EDI) assessment tool.

| **Stage of project (linked to flow chart)** | **Activity** | **Reviewed?** | **Notes on how this was considered within this project, and decisions made.**  **If activity not considered, please briefly add details on why this was not possible in this particular evaluation.** |
| --- | --- | --- | --- |
| Building initial team | 1. Ensure evaluation teams include a diverse range of team members *[e.g. gender, age, ethnicity, seniority and other characteristics*] | YES | RSET team is diverse in terms of gender, age, ethnicity and seniority. This is reflected in this project team. |
|  | 1. Ensure project steering groups include a diverse range of evidence users and healthcare professionals *[e.g. gender, age, ethnicity, seniority, role and other characteristics]* | YES | Good range of expertise/specialty, gender, ethnicity. |
|  | 1. Ensure project PPIE panel includes a diverse range of patients/carers. *[e.g. gender, age, ethnicity, experience and other characteristics]* | YES | Range of gender, age, ethnicity among the public contributors. |
| Discovery and scoping | 1. Consult with **PPIE group** and **evidence users** (through scoping discussions) to understand EDI implications of both the intervention and our evaluation. | YES | Yes – this was a focus of discussions at   - initial meetings with PPIE members - stakeholder workshops for Phase 1 and 2 - stakeholder engagement during scoping for Phase 1 and 2, including meetings and e-mail consultation - public contributor on the advisory panel |
|  | 1. During scoping conversations, the way in which PPIE members and evidence users are consulted should be adapted appropriately for each audience. *For example, it may be necessary to provide information in alternative formats other than standard text if people need or prefer that.* | YES | We asked PPIE workshops attendees about their preferences around sharing of information or opportunities to feed back.  For Phase 2, these discussions also focused on approaches to recruiting patients and carers, and the structure and content of patient and carer interview topic guides. |
|  | 1. Use EDI published frameworks (e.g. Health Inequalities Assessment Tool;^101^ INCLUDE framework;^99^ toolkit for increasing participation of Black, Asian and Minority Ethnic (BAME) groups in health and social care research).^102^   *These frameworks will help ensure that our projects are designed to be inclusive and address appropriate questions (e.g. considering underserved groups and wider protected characteristics, barriers to inclusion and steps to overcome barriers).* | YES | We used the Health Inequalities Assessment tool to ensure thorough consideration of EDI throughout all stages of the project. For example, this helped to identify potential EDI issues relevant to this topic including bias in the AI tool testing, trust and location related inequalities, and accuracy of AI for those with different characteristics or conditions. |
| Stakeholder engagement | 1. Discuss project with **project PPIE group and project advisory group** and ensure projects address EDI issues, including: 2. Whether and how different communities were involved in planning, 3. Whether and how research approaches accommodate and measure potential impact on EDI considerations, 4. Evaluating the intervention’s impact on access, patient experience, engagement, and outcomes across different communities) 5. work with stakeholders to reflect on progress of the work and ensure our findings address implications for EDI. | PARTLY | We discussed project specific EDI issues with our PPIE members, Project Advisory Panel, stakeholder workshop, and RSET Stakeholder Advisory board. |
| Data collection – **focus** | 1. Develop research questions that address any issues of inequalities, inequities and disparities, as appropriate. | YES | Research questions consider implications of AI for EDI. |
|  | 1. Identify how any relevant quantitative data reflects population diversity. | YES | Flowing from Phase 1, quantitative analyses will attempt to capture data in relation to population diversity where available. |
| Data collection – **site recruitment** | 1. Select study sites to represent a range of characteristics wherever possible (including geography, ethnicity, rurality, socioeconomic status). | YES | See sampling strategy, in terms of geographic location (including socio-deprivation, inequalities and clinical pathways). Final sample included majority of networks – good range of characteristics. |
| Data collection –**participant recruitment** | 1. Plan to recruit samples of patients, carers and staff that include a range of participants of different ages, gender, ethnicities, living circumstances, educational qualifications, work situations, and disability. | YES | See sampling: for our patient and carer interviews, we will seek to recruit patients across a range of characteristics, including health outcome following review of scan (and therefore care pathway), and factors relating to socio-demographic characteristics (e.g. gender, age, ethnicity, disability) |
|  | 1. Where possible, compare our study sample characteristics to national or local populations accessing and delivering services (e.g. see^15^) | YES | Where available, we will be drawing on national datasets, e.g. HES and DIDS. |
|  | 1. To support recruitment of a range of participants, consider the following strategies and other strategies as necessary (depending on appropriateness for each evaluation and conversations with stakeholders and PPIE panel): 2. Translating research materials into a range of languages or different formats where appropriate, e.g braille, or British sign language 3. Community outreach to recruit participants (e.g. through patient and staff organisations) 4. Offer different modes of data collection (e.g. in person, telephone or online for interviews/focus groups/observations and online or paper surveys), 5. Offer different options for participation (e.g. participant only, participant and carer, or carer only interviews) 6. Offer translation services to facilitate interviews. 7. Ensuring participants have reasonable access to participating in the study   *It may be helpful to look at the* [*NIHR’s definition of underserved communities*](https://www.nihr.ac.uk/documents/improving-inclusion-of-under-served-groups-in-clinical-research-guidance-from-include-project/25435) *when thinking about how best to recruit different groups* | Not applicable | We will be recruiting patients via hospital trusts/services, as we wish to interview people who have undergone diagnostic scans supported by AI. However, our sample has been designed to capture perspectives of less well served communities.  We will ask staff/research nurses to recruit across a range of patient characteristics (see above).  We have capacity to translate our recruitment documentation in the event that potential interviewees require this, and we will explore the option of offering an interpreter service for interviews.  All recruitment documentation will be available in hard copies (if required), as well as electronically and compatible with screen reader software.  We will also offer potential interviewees the option of discussing the research with a member of the team in the event that they require further information. |
| Analysis | 1. Use frameworks to support equity-focused analysis where appropriate (e.g. EquIR).^59^ | YES | We will incorporate EQUIR constructs into RAP sheets. |
|  | 1. If available, analyse data to identify differences in service use and outcomes across different population groups | YES | The quantitative workstream plan to request DID data with nested demographic variables to allow us to consider intersectional identities as opposed to each demographic variable in isolation of each other.  For the Health Economic workstream, given the completeness and granularity of the available data, it is unlikely that independent economic modelling for different demographic groups will be possible. In reporting our findings, we will consider the potential for differential impacts on patient groups in the context of relevant issues raised across other workstreams.  For the qualitative workstream, we are employing variation sampling, but given numbers it will not be possible to perform formal comparisons between or make generalisations about different patient characteristics. However, we will seek to identify issues specific to certain patient groups’ experiences (e.g. around communication for people who do not have English as a first language). |
|  | 1. Work with stakeholders **(project advisory group and PPIE)** to reflect on progress of the work and ensure our findings address implications for EDI | YES | EDI as a standing agenda item on weekly team meetings and advisory group meetings. |
| Dissemination | 1. Work with stakeholders **(project advisory group and PPIE)** to develop and agree a dissemination and mobilisation strategy that supports sharing findings with all relevant audiences (including diverse and underserved communities). | YES | Yes – have consulted dissemination strategy with wide range of stakeholders as part of peer review and meetings, including with third sector organisations and PPIE representatives. |
|  | 1. Work closely with stakeholders **(PPIE panel, and project advisory group)** to share findings (e.g. as co-authors and co-presenters). | YES | We continue to work closely with stakeholders (PPIE members, project advisory group) to develop dissemination outputs. |
|  | 1. If quantitative analyses of differences between population groups has not been possible, make recommendations about how to enable this for future evaluations. | YES | Again, we will explore this in our analyses (including our workshops with national and service-level stakeholders) and reflect on this in our resulting reports. |
| *Note:* Throughout all our activities, we will be facilitated by guidance on effective EDI. [e.g. National Institute for Health and Care Research. Equality, Diversity and Inclusion Toolkit 2022. [Retrieved 09/12/2022 from <https://www.rdsresources.org.uk/edi-toolkit>] */* NIHR EDI strategy (2022-2027*)* [*https://www.nihr.ac.uk/documents/equality-diversity-and-inclusion-strategy-2022-2027/31295*](https://www.nihr.ac.uk/documents/equality-diversity-and-inclusion-strategy-2022-2027/31295) | | | |

Note: This tool was completed on 4^th^ February 2025 during study protocol development phase. We will review the tool again during the data collection and analysis phase.
