# Supplementary material for: Implementation, Experiences, Impact, and Costs of Artificial Intelligence in Chest Diagnostics: Protocol for a Mixed Methods Evaluation
Source: JMIR Res Protoc. 2025 Oct 31;14:e81421. doi: 10.2196/81421 (PMC12619010; doi:10.2196/81421)
Supplement: Multimedia Appendix 2 [file resprot_v14i1e81421_app2.docx]

## **Topic Guides for Semistructured Interviews**

**Topic Guide** – Staff Interviews

*[Introduction to the project and a summary of the topics covered in the interview]*

| **Topic** | **Main question/s** | **Follow-up questions/prompts** |
| --- | --- | --- |
| JOB ROLE  & DEMOGRAPHICS | - Can you briefly tell me about your current role? - Job role - Location - Length of time in role | - What are your main responsibilities? - Have you had any previous experience/ involvement with AI? - How have you been involved with implementing AI for diagnostics in your organisation? Can probe AIDF specific examples if needed |
| VIEWS ON REASONS FOR IMPLEMENTING AI  (Decision to change and implementation approach – framework) | 1. [briefly, in a nutshell] What do you think is driving the implementation of AI in healthcare and your local organisation? | - What do you think about using AI for diagnostics (whether they support the use of AI in real-world settings/clinical practice) - Anticipated impact - How can AI help and/or hinder the diagnostic process? |
| **HOW AI TOOL IS BEING USED** – FUNCTIONS AND USE IN PATHWAY  (Decision on which model to implement/model selection – framework) | 1. Could you please [briefly] describe the AI tool being used in your organisation? | - What is the purpose of the AI tool (e.g., are you using it for pathway prioritisation, detection, second reader etc) - Why they chose to go with this model - Are these platforms only used in radiology? |
|  | 1. Could you please [briefly] walk us through how you are using the AI tool in practice (from initial referral, scan, results, outcome)? | - Who receives the initial referral? - Who uses the AI tool (just radiology staff, wider clinical staff)? - Is there an order to who views the scans (e.g., do staff view the AI before or after they view the scan and make a diagnosis) - What happens after the results are received? - How is the information stored/transferred? - Have these processes all remained the ***same***? Or have any changed/ been adapted as AI has continued to be used? - Is the way AI is being implemented the same as the way it was intended to be implemented? |
|  | 1. Who is AI used for? Who is eligible? | - What are the eligibility criteria? Has this remained the same of changed? |
|  | 1. Could you please tell us about the diagnostic care pathway and describe how has AI been integrated into this pathway? | - Where does the AI fit in the diagnostic pathway? - How has the pathway been changed to enable AI to be integrated? - What did the pathway look like ***before*** and ***after*** the implementation of AI? Some examples? - Are the interfaces between the AI and NHS systems compatible? Or has there been resource required to support the changes? - Have you noticed changes in patient outcomes (e.g., reduced diagnostic errors, earlier detection) since using AI? - Are there metrics used to measure clinical impact? For example, reductions in delayed diagnoses, or adverse events - How has the AI tool impacted turnaround times for chest diagnostic results? Are there measurable reductions compared to pre-AI processes? |
|  | 1. How, if at all, are you informing patients about the use of AI? [*must ask all interviewees] | - Examples of communication - Rationale for informing patients and/or not informing patients – and implications of this - Who was involved in this decision making? Will this decision-making process continue, or potentially change? - Is the AI report shared with patients when they get given the report/results of their scan? - What are your thoughts about patients having adverse reactions to AI (if they know about it being used)? How could this be mitigated against? |
|  | 1. Have there been any adaptions in the use of AI over time? | - Any changes to service model and updates based on any feedback from the clinical teams? - Any changes due to software updates (e.g., the supplier updating their tool) - is there training/comms about this if it happens - Any changes to governance - If so, could you provide some examples of this? - Are there plans for any of these adaptations/changes to be long-term? |
| **IMPLEMENTATION** – KEY PROCESSES (E.G., PLANNING AND FACILITATION)  (Implementation outcomes and approach, adoption – framework) | 1. How did you (or your organisation) plan for the implementation of AI? | - How do you feel this impacted implementation? - Who was involved in the planning and facilitation? - Did planning help/ hinder? - How has AI been communicated to staff – to raise awareness? - Is there anything you would have done differently? Examples of their plans? - What baseline data was collected before AI implementation (e.g., pre-AI costs, diagnostic error rates, or time metrics)? How is this being compared to post-AI data? |
|  | 1. Could you walk us through the process of implementing AI? | - What were the key processes? - How did it work? - Which groups were involved in supporting the process? - How well is the AI being adopted? - How has the implementation of AI impacted departmental budgets? - Impact of change in context (NHSE and ICB cuts) |
|  | 1. What has helped/ hindered when implementing AI? | - Relationships with/between services, networks (e.g., shared learning and collaboration) and suppliers - Training and knowledge about use of AI - AI champions/experts - Staff capacity/resources - Leadership and governance - Staff views (e.g., support and engagement vs resistance) - Support from NHSE and/or supplier |
|  | 1. What has made implementing AI more challenging? | - Support or lack of (lack of relationships between services, networks and suppliers, troubleshooting) - Lack of training - Lack of leadership and governance - Unclear communication - Challenges with staff capacity and resources - Staff views (e.g., support and engagement vs resistance) - Lack of AI champions/experts - Impact of change in context (NHSE and ICB cuts) - What causes the challenges? Is it IT infrastructure? |
| STAFF EXPERIENCES OF USING THE AI TOOL  (Implementation outcomes – framework) | 1. What are your experiences of using the AI tool in practice? | - What are your views on using the AI tool? - How often are you using the AI (e.g., do you always refer to the AI report if being used as a second reader)? - Positive experience vs negative experience (or both) - Key barriers and enablers - Are you comfortable using the AI tool? - Do you understand the AI tool? - Do you trust the AI tool? - Any changes they would make to how the AI tool is being used? |
|  | 1. Could you describe any training you have received during the implementation of AI to help with using the technologies in practice? | - What did you think of the training? - Who provided the training? - Did/do you feel prepared to use AI following the training? - Would you change anything about the training? |
|  | 1. What has helped/hindered when using the AI tool in practice *(beyond implementation – focus on clinical use here)*? | - Relationships with/between services, networks (e.g., shared learning and collaboration) and suppliers - Training and knowledge about use of AI – clear understanding of the AI tool/what it is being used for and how they should be used - AI champions/experts - Staff capacity/resources - Leadership and governance - Staff views (e.g., support and engagement vs resistance) - Support from NHSE |
|  | 1. What has been challenging about using the AI tool in practice *(beyond implementation – focus on clinical use here)*? | - Support or lack of (lack of relationships between services, networks and suppliers) - Lack of training - Lack of leadership and governance - Unclear communication - Challenges with staff capacity and resources - Staff views (e.g., support and engagement vs resistance) - Lack of AI champions/experts |
| STAFF PERCEPTIONS OF IMPACT  (Implementation outcomes – framework) | 1. In your experience, how, if at all, does AI impact how you provide diagnostic care? Do these outcomes algin with the intention with which AI was adopted? | - If so, could you provide some examples? - *What does AI impact* (e.g., time to report, speed through diagnostic pathway, improved accuracy, identifying missed diagnoses etc)? - Positive/negative impact - How has the AI impacted staff productivity, such as time spent on diagnostic processes or workload distribution? If so, how has this been quantified? - Are there specific examples of how AI has streamlined workflows or reduced manual tasks? - Has the adoption of the AI required reallocation of resources, such as hiring additional staff or retraining existing staff? If so, how are these costs reported? - Has the AI tool led to cost avoidance in areas such as unnecessary imaging, reduced testing, or fewer diagnostic procedures? Can you share specific examples? - Are there measurable reductions in consumable use (e.g., contrast imaging) due to AI-assisted diagnostics? |
|  | 1. What do you think are the benefits of using AI for chest diagnostics? (*must ask all interviewees) | - *Impact on patients:* e.g., quicker diagnosis, reduction in false negatives and any reduction in cancer stage at diagnosis vs being unaware of AI, changes to their pathway (e.g., being de-prioritised) - *Impact on staff:* e.g., reduced workload/ decision support, training and learning opportunities vs increasing workload, challenges with capacity to implement - *Impact on the wider healthcare system:* e.g., helping to address wider challenges such as increased demand for imaging vs creating additional challenges or not changing demand |
|  | 1. Do you have any concerns about using AI in practice? Could there be any disadvantages or unintended consequences? | - Ethical implications (e.g., responsibilities, data privacy etc) - The bias in AI algorithms/the data the tools are trained on - Potential misuse of AI - AI making errors - Time taken for staff to get used to AI - Changes to communicating with patients - Changes in who delivers ‘news’ (outcomes) to patients - Will there be an impact later down the pathway? E.g., if people get to treatment more quickly will they just get stuck at the next phase - *For procurement teams/leads (or other roles if relevant):* Where does the data go? How is the data used? Who owns the rights to the data? |
|  | 1. Which factors do you think are likely to have influenced impact the most? | - Function of AI - IT infrastructure - Patient profile |
|  | 1. How do you think that implementation approaches and service models have influenced the impact of AI? | - Service delivery - Patient outcomes - Impact of change in context (NHSE and ICB cuts) |
| IMPLICATIONS FOR EDI  SUSTAINABILITY  (Implementation outcomes – framework) | 1. In your experience, could using AI have any implications for equality, diversity and inclusion (e.g., can AI impact different population groups)? (**must ask all interviewees) | - Could you give examples of impact/implications for inequalities? - Are you monitoring the impact on different groups? If so, how? - Are there standardised procedures for assessing biases in AI outputs (e.g., providing information in different languages)? - Any scans where there have been difficulties in getting a good reading (e.g, false negatives/positives, invalid scans) – do any relate to demographic characteristics? |
| DATA MONITORING AND EVALUATION  (Implementation outcomes – framework) | 1. Are there any ongoing issues with collecting data for the NHSE benefits metrics? | - If there were problems before, have they been improved? - Do you have any thoughts about data quality? |
|  | 1. Is there a local evaluation (in your network/trust)? | - Who is doing the evaluation? - How has the evaluation progressed? - What does your local evaluation involve? Which data are you using? - Are you aware about suppliers doing post-market surveillance? If so, what is required from them to facilitate this? Is it time consuming? |
|  | 1. Any relevant data questions arising from workstreams 2 and 3 | - *Question for strategy leads/ senior decisionmakers only:* Is your Trust preparing a local business case for funding to continue the AI tool? (eg local Trust funding or network-level funding)   - If yes - what are the timeframes for the business case, and where might these independent evaluation findings fit in? |
| FUTURE USE AND SUSTAINABILITY  (Implementation outcomes – framework) | 1. The use of AI is increasing in the NHS and AI is often viewed as a ‘transformative solution’. Do you think that AI *will continue to be used* for chest (and diagnostics more broadly) in the future? | - If yes, what might this look like? - What is important to consider moving forward? - Future barriers/ facilitators - Impact of change in context (NHSE and ICB cuts) - Key reflections – perceived value of having a national programme |
|  | 1. Do you think AI *should be used* in the future? | - If yes/no, why? And how? And in which ways? Are there any red lines/ areas where AI should definitely not be used? - What is important to consider moving forward? - Future barriers/ facilitators - Do you think it can be used in the long-term (e.g., be a sustainable part of care)? - How is feedback from clinicians used for the further/future development or improvement of the AI tool? - Impact of change in context (NHSE and ICB cuts) - Key reflections – perceived value of having a national programme |
| WRAP UP | Is there anything else you would like to add that we haven’t discussed? |  |
| DISSEMINATION LIST | Would they like to be added?   - Yes - No |  |

**Topic Guide** – Supplier Interviews

*[Introduction to project* *and a summary of the topics covered in the interview]*

**Note: We appreciate that certain matters might be commercially sensitive/confidential*

| **Topic** | **Main question/s** | **Follow-up questions/prompts** |
| --- | --- | --- |
| JOB ROLE, ORGANISATION AND PRODUCT INFORMATION | 1. Can you tell me a bit about your organisation and current role? | - What are your main responsibilities? - Have you had any previous involvement with AI? - How have you been involved with implementing AI for diagnostics in your organisation? Can probe AIDF specific examples if needed |
|  | 1. How long have your organisation been working with the healthcare sector and what have been your experiences of this? | - Just NHS or other healthcare systems? - Any strategic benefits/disbenefits to working with healthcare/NHS (e.g., MHRA regulation)? Are there any factors which have made other systems easier/more difficult to work with? - Long-term plans for supplying AI within healthcare |
|  | 1. Could you describe the AI tool that you are providing for the networks and trusts as part of AIDF? | - What is the AI tool being used for? (e.g., second reader, prioritisation) |
|  | 1. If through implementing AI tools in the NHS your tool can be enhanced/ improved, who owns the intellectual property? | - *Examples:* if clinicians are feeding back on their experiences, their training, process of local integration etc |
|  | 1. How is the AI tool intended to be used *(and being used)* in practice? | - Do you know that AI is used across multiple trusts How is the AI being used in practice? Are there any differences across multiple networks/trusts? - How does it work? - How has it been tested/trained (EDI considerations)? For specific local contexts? - Are there standardised training processes for AI across networks? - How is it being implemented into clinical pathways? - Any adaptations for networks or trusts? |
|  | 1. How are your AI tools implemented across different trusts (and networks if supplying to multiple networks)? | - What are the similarities and differences? - What is working well/not so well from your perspective? - Is where anything which could make things work better (from NHS or supplier side, or both) |
| VIEWS ON REASONS FOR IMPLEMENTING AI | 1. What do you think is driving the implementation of AI? | - Agreement/disagreement with proposed use (whether they support the use of AI in clinical practice) - Anticipated impact - How can AI help and/or hinder the diagnostic process? |
| EXPERIENCES OF EARLY DEPLOYMENT | 1. Can you walk me through your experiences of the early deployment phase? | - What have been your key responsibilities? - How have you been engaging with networks and trusts? How have you monitored and supported the deployment phase? - What worked well - What didn’t work well - Are there key lessons you will take forward? Anything you might change when supplying AI in healthcare settings in the future? - Impact of change in context (NHSE and ICB cuts) |
|  | 1. What has been challenging for you as a supplier during this phase? | - Impact of these barriers - Staff views (e.g., support/ engagement or resistance) - How were these addressed? - Is there anything that you would change? - Impact of change in context (NHSE and ICB cuts) |
|  | 1. What has helped you as a supplier during this phase? | - Impact of these facilitators - Staff views (e.g., support/ engagement or resistance) |
| EXPERIENCES OF IMPLEMENTATION | 1. Can you walk me through your experiences of implementation – from early deployment until now? | - What have been your key responsibilities? How have these changed and evolved? - How have you been engaging with networks and trusts? - What worked well - What didn’t work well - Are there key lessons you will take forward? |
|  | 1. What has been challenging for you as a supplier during implementation? | - Impact of these barriers - How were these addressed? - Staff views (e.g., support/ engagement or resistance) - Impact of change in context (NHSE and ICB cuts) |
|  | 1. What has helped you as a supplier during implementation? | - Impact of these facilitators - Staff views (e.g., support/ engagement or resistance) |
|  | 1. What have been your experiences of integrating your AI tool into local IT systems? | - Have you found this challenging and/or easier than anticipated? - Has this depended on local IT capacity? - Barriers and facilitators - Impact of IT capacity and infrastructure - What costs are associated with integrating the AI tool with existing IT systems (e.g., additional costs associated with customising the tool for different NHS trusts, ongoing costs for maintaining and upgrading the AI tool)? |
| SUPPORT WITH IMPLEMENTATION | 1. How have you been supporting networks and trusts throughout AI implementation? | - Some examples: e.g., attending meetings, running training etc - How has this helped implementation? - Was there anything else which could have been done? - Are there lessons you will take forward? |
| PERCEIVED IMPACT OF AI TOOLS | 1. From your point of view, how, if at all, does AI impact diagnostic care? Do these outcomes algin with the intention with which AI was adopted? | - If so, could you provide some examples? - *What does AI impact* (e.g., time to report, speed through diagnostic pathway, improved accuracy, identifying missed diagnoses etc)? - Positive/negative impact |
|  | 1. What do you think are the benefits of using AI for chest diagnostics? | - *Impact on patients:* e.g., quicker diagnosis, reduction in false negatives and any reduction in cancer stage at diagnosis vs not being informed, changes to pathway (e.g., being de-prioritised) - *Impact on staff:* e.g., reduced workload/ decision support, training and learning opportunities, vs increased workload, impact on capacity - *Impact on the wider healthcare system:* e.g., helping to address wider challenges such as increased demand for imaging vs negative impact on systems in terms of workload - Impact for you as a *supplier*? - How, if at all, are you monitoring impact as suppliers? |
|  | 1. Do you have any concerns about using AI in practice? Could there be any unintended consequences? | - Ethical implications (e.g., responsibilities, data privacy etc) - AI making errors - Time taken for staff to get used to AI - Changes to communicating with patients - Changes in who delivers ‘news’ (outcomes) to patients |
|  | 1. Which factors do you think are likely to have influenced impact the most? | - Function of AI - IT infrastructure - Patient profile |
|  | 1. How do you think that implementation approaches and service models have influenced the impact of AI? | - Service delivery - Patient outcomes |
|  | 1. In your experience, could using AI have any implications for equality, diversity and inclusion (e.g., can AI impact different population groups)? | - Could you give examples of impact/implications for inequalities? - Are you monitoring the impact on different groups? If so, how? - Any scans where there have been difficulties in getting a good reading (e.g, false negatives/positives, invalid scans) – do any relate to demographic characteristics? |
| DATA MONITORING AND EVALUATION | 1. Can you quantify the impact of AI? | - How are you able to quantify impact? - What evidence do they have on this?   Post-market surveillance:  Is this being done as part of data collection and evaluation? How often is this happening? Is it useful?   - Do they have any other evaluations ongoing? - Do they have any obligations to collect data – are these obligations clearly defined? - What is the process from obtaining data from clinical teams? |
|  | 1. Are you able to share any of their data with us? | - Details on what data they are able to share/what data they cannot share and why |
| FUTURE OF USING AI | 1. The use of AI is increasing in the NHS and AI is often viewed as a ‘transformative solution’. Do you think that AI *will continue to be used* for chest (and diagnostics more broadly) in the future? | - If yes, what might this look like? - What is important to consider moving forward? - Future barriers/ facilitators - Impact of change in context (NHSE and ICB cuts) - Key reflections – perceived value of having a national programme |
|  | 1. Do you think AI *should be used* in the future? | - If yes/no, why? - What is important to consider moving forward? - Future barriers/ facilitators - Impact of change in context (NHSE and ICB cuts) - Key reflections – perceived value of having a national programme |
|  | 1. Do you think that AI can be used to support diagnostics in the long-term? | - Do you think AI is a sustainable tool? - How might AI be involved in long-term care? |
| WRAP UP | Is there anything else you would like to add that we haven’t discussed? |  |
| DEMOGRAPHICS | - Job role/title - Supplier - Length of time in role |  |
| DISSEMINATION LIST | Would they like to be added?   - Yes - No |  |

**Topic Guide** – Patient representative group Interviews

- Introduce the researcher and project
- Outline what we will discuss in the interview
- Say the interview will last approximately 45 minutes to one hour, or less, depending on how much they would like to say
- Emphasise that there are no right or wrong answers and taking part is completely voluntary, and that they are free to withdraw at any time
- Say that the interview will be audio recorded and professionally transcribed, but findings will be fully anonymised (names, places)
- Thank for providing consent ahead of interview, or take verbal consent – check still happy to participate
- Turn recorder on
- Check if they are a patient or carer (if not known from recruitment and eligibility)

***Part 1 –*** *Experiences of diagnostic care*

*[Introduction to project, Part 1 of the interview and summary of what will be covered in the interview]*

| **Topic** | **Main question/s** | **Follow-up questions/prompts** |
| --- | --- | --- |
| ABOUT THEM | 1. Please could you tell me a bit about yourself? | - Occupation/role in organisation |
| The organisation/group | 1. What is the role of your organisation in supporting patients and carers | - Role (e.g. advocate, service provider, commissioner) - Relationship with NHS services - Past interactions with NHS organisations |
| BARRIERS AND FACILITATORS | 1. Are you aware of patients and carers you represent experiencing any challenges or barriers when receiving their diagnostic care? | - If yes, could you provide some examples of these barriers? - If no, what worked well when receiving your diagnostic care? - How might these barriers be addressed? |
|  | 1. Did anything help with receiving their diagnostic care? | - If yes, could you provide some examples of how these factors helped you? |
| EXPERIENCES OF CARE | 1. Overall, how would you describe patients’ experiences of receiving diagnostic care? | - Positives and negatives |
|  | 1. Is there anything which could have been improved? | - Recommendations for future care |

**Part 2** – *Views and perceptions on the use of AI*

*[Explain now moving onto part 2 and briefly describe what will be covered in the interview - their views towards AI]*

| **Topic** | **Main question/s** | **Follow-up questions/prompts** |
| --- | --- | --- |
| KNOWLEDGE AND UNDERSTANDING OF AI | 1. How would you describe your current knowledge and understanding of AI? | - Has your organisation consulted with your patient community or patient groups/ done any research on AI with your members? - Where have you learned about AI? E.g., which sources of information have you used – (like social media, news articles etc) - Would you like to know more (or less) about AI? |
|  | 1. What do you know about how AI is being used in healthcare? | - Have you heard about AI being used in healthcare? - Do you know *how* AI is being used in healthcare – any examples (e.g., to assist with diagnosis, to assist with more administrative and/or routine tasks etc)? |
| VIEWS ON AI BEING USED IN CARE | 1. What do you think about AI being used in healthcare more broadly? | - Do you have an organisational stance on the use of AI in healthcare, and/or diagnostics specifically? - Do you agree with the use of AI? - Do you have any concerns about using AI in healthcare? |
|  | 1. Are you aware of any AI currently being used in diagnostic care? If so, could you describe some examples? | - How did you know that AI is currently being used in care? - Do you think AI should be used? |
|  | 1. What do you think about staff members looking at a scan with AI input (e.g., highlight areas of concern) to help when making their decisions? | - What do you think is good or bad about AI being used like this? For patients, clinicians and NHS/wider system - Is there anything that would influence your perspective? *Prompts:*   - How the use of AI is communicated to you?   - Staff available at the hospital to review scans?   - Length of time the specialist staff reading your scan have been qualified for?   - Amount of training the staff have had to use the tool?   - How severe the condition is?   - The number and type of scans used to train the AI tool (e.g, scans on people with different conditions and backgrounds)? |
|  | 1. What do you think about AI being used **to prioritise X-rays or CT scans** to be reviewed by specialist staff (e.g., if the AI suggests there might be signs of cancer or other health conditions then these cases are checked by specialist staff more quickly)? | - What do you think is good or bad about AI being used like this? For patients, clinicians and NHS/wider system - Is there anything that would influence your perspective? *Prompt:*   - How the use of AI is communicated to you?   - Staff available at the hospital to review scans?   - Length of time the specialist staff reading your scan have been qualified for?   - Amount of training the staff have had to use the tool?   - How bad the condition is?   - The number and type of scans used to train the AI tool (e.g., scans on people with different conditions and backgrounds)? |
| Introduction to example scenarios  We’re now going to look at some example situations that could happen when AI is used to help review chest X-rays.  Before we go through these examples, it’s worth knowing that:   - there is some evidence to show that AI tools have a similar accuracy to a human radiographer (i.e. there is a similar level of error) - the safety of the AI tools are regularly checked - situations like these can also happen now, even when AI isn’t used. For example, it’s common for some people to have extra tests that then show nothing serious, and sometimes important findings are only spotted a little later. In these examples, the AI is simply there to help the healthcare team decide which X-rays to look at sooner — but a trained clinician will always make the final decision about your care.   In each example, I’ll explain what happens and then ask you how you might feel in that situation and what might influence your view. | | |
| VIEWS ON POTENTIAL **OUTCOMES** OF AI USE  *[True positive;*  *False positive;*  *False negative;*  *True negative]* | 1. OPTIONAL SCENARIO 1 (TRUE POSITIVE) The AI tool does a first reading of a patient’s X-ray, and highlights possible signs of cancer. The X-ray is then prioritised, and looked at by specialist staff sooner. The specialist agrees with the AI and recommends a CT scan, which gives more detailed information and confirms findings suspicious of cancer. AI has helped prioritise the initial specialist review so that the patient could then get diagnosed and treated more quickly. | OPTIONAL SCENARIO   - How would you feel about your scan being prioritised in this situation? - How much trust would you have in the use of AI in chest diagnostics, in this situation? (on a scale of 1-5, 5 being full trust) - Would you want to be told that AI had been used to help the specialist review your scan, or would that not be important to you as long as a specialist was making the final decision? |
|  | 1. SCENARIO 2 (FALSE POSITIVE) The AI tool does a first reading of a patient’s X-ray, and it highlights possible signs of cancer. The X-ray is then prioritised to be looked at by specialist staff sooner. The specialist agrees with the AI and recommends a CT scan, which gives more detailed information. The CT scan suggests the patient has no signs of cancer or other health conditions, and the patient is reassured by their clinician (eg GP). | - How would you feel about having an additional CT scan in this situation? - How much trust would you have in the use of AI in chest diagnostics, in this situation? (on a scale of 1-5, 5 being full trust) - If we told you that going for additional scans happens quite often in standard NHS care without AI, would this change your view?* - Would you want to be told that AI had been used to help the specialist review your scan, or would that not be important to you as long as a specialist was making the final decision?   **Note: In NHS cancer pathways, it is common for patients to have further tests that do not confirm cancer — e.g. only about 3% of patients referred on a suspected lung cancer pathway are found to have the disease. This process helps ensure that potential cancers are not missed.* |
|  | 1. SCENARIO 3 (FALSE NEGATIVE) The AI tool does a first reading of a patient’s X-ray, and it reports finds no signs of cancer or other health conditions. This means that it is not flagged by AI for urgent specialist staff review, and is looked at by staff in the usual timeframe. When specialist staff look at the X-ray, they identify suspected cancer and recommend a CT scan, which confirms findings suspicious for cancer. The patient then goes for more tests to confirm the cancer diagnosis. In this case, AI missed the opportunity to fast-track the initial specialist review. | - How would you feel about your scan not being prioritised in this situation? - How long a delay to the clinician’s report would be acceptable to you? (e.g. up to 3 days, 1 week, 2 weeks?) - How much trust would you have in the use of AI in chest diagnostics, in this situation? (on a scale of 1-5, 5 being full trust) - Would you want to be told that AI had been used to help the specialist review your scan, or would that not be important to you as long as a specialist was making the final decision? |
|  | 1. OPTIONAL SCENARIO 4 (TRUE NEGATIVE) The AI tool reads a patient’s X-ray, and it finds no signs of cancer or other health conditions. This means that it is not flagged for urgent specialist staff review, and is looked at by staff in the usual timeframe. When specialist staff look at the X-ray, they agree there are no suspicious findings and issue a radiology report. The report explains the X-ray is normal but advises the clinician to review the patient in 6 weeks and consider further investigation if symptoms persist. This might take longer to report than the X-rays AI has prioritised, but is still checked within the hospitals target reporting times for normal X-rays. | - How would you feel about your scan not being prioritised in this situation? (Would it matter to you if your results took slightly longer because other, more urgent scans were prioritised?) - How much trust would you have in the use of AI in chest diagnostics, in this situation? (on a scale of 1-5, 5 being full trust) - Would you want to be told that AI had been used to help the specialist review your scan, or would that not be important to you as long as a specialist was making the final decision? |
| INVOLVEMENT IN PROCUREMENT AND DEPLOYMENT | 1. Have you or your group/organisation been involved in procurement or deployment of AI tools? | - Role/activities - Time/resources committed - Obstacles/enablers - Lessons |
|  | 1. How might NHS organisations work with your group/organisation in future? | - Role activities (engagement, consultation, codesign, comms, quality checking) - What would be needed, e.g. timing, resources, engagement - What might be obstacles/enablers |
| INFORMATION GOVERNANCE | 1. If AI is used in diagnostic care, do you think that patients should be informed? | - Why do you think informed consent from patients is and/or is not important? |
|  | 1. How should healthcare providers be explaining the use of AI to patients? | - How much *detail* should be included? - What communication approaches do you think are most effective for building public awareness and trust? - How should communications be tailored or bespoke to any specific patient groups or communities? |
| BENEFITS OF USING AI | 1. What do you think are the benefits of using AI to assist with diagnostic care? | - How can AI benefit patients? - How can AI benefit staff? - How can AI benefit hospitals or healthcare systems such as the NHS? - Provide examples |
| CHALLENGES OF USING AI | 1. What do you think are the challenges and/or barriers to using AI to assist with diagnostic care? | - What might be the challenges for patients? - What might be the challenges for staff? - What might be the challenges for hospitals or healthcare systems such as the NHS? - Provide examples |
| POTENTIAL IMPACT OF AI AND UNINTENDED CONSEQUENCES | 1. If AI is used in diagnostics, what impact do you think it could have? | - Impact for *patients* - Impact for *staff* - Impact for *hospitals or healthcare systems* such as the NHS? |
|  | 1. Do you think that there could be any unintended consequences of using AI for diagnostics? | - Provide examples of these - What could be the impact of these unintended consequences? |
| VIEWS ON POTENTIAL AUTONOMOUS AI REPORTING IN FUTURE (I.E. NO HUMAN REVIEW) | 1. In future, if the law was changed, AI might be used to review and report a patient’s X-ray without any human involvement (ie specialist staff checking it). This would only happen if it can be proved that AI tools are as good as or better than specialist staff at reading X-rays.   Any such changes would only be made gradually and carefully in the NHS: starting with using AI tools only on X-rays that are very likely to be normal, with regular safety checks by specialist healthcare staff. | - How acceptable would this use of AI be to you? - How much confidence/trust would you have in this use of AI? - What do you think about the use of AI in this way, for different types of patients? (e.g. ethnicity, gender, age, condition) - Who should be responsible if errors occur? (e.g. hospital clinicians, suppliers, regulators, government) - What might influence your view? |
| FUTURE USE | 1. What do you think about AI being used in the future? | - Do you think that AI should be used in the future, and why? - What might be future barriers and facilitators to using AI in future? - What might concern you about AI being used in the future? - What do you think is good about using AI in the future? - What do you think about patient data being used to help improve AI? - How would you feel about AI tools learning to be safer and more accurate based on yours (and other peoples scans, with consent)? |
| WRAP UP | 1. Is there anything else you would like to add that we haven’t discussed? |  |
| DEMOGRAPHICS | - Sex - Gender - Age - Ethnicity - Language (e.g. is English their first or second language) - Education level - Condition/s (which required the diagnosis) - Postcode / location - Level of familiarity/understanding of AI in general |  |
| DISSEMINATION LIST | Would they like to be added?   - Yes - No |  |

**Topic Guide** – Patient and Carer Interviews

- Introduce the researcher and project
- Outline what we will discuss in the interview

*Hello, my name is [add here] and I am a researcher working as part of NIHR RSET, a team based at UCL, the Nuffield Trust, and University of Cambridge. We are looking to understand patient experiences of going for a chest scan like an X-ray or CT scan (their diagnostic care).*

*In the first part of this interview, we will briefly ask you about your experiences of going for an X-ray and/or CT-scan, getting results, and what happened afterwards.*

*In the second, longer part of the interview, we will ask about your views on the current use of artificial intelligence in chest diagnostics. When we talk about ‘artificial intelligence’ or ‘AI’ what we mean by this are computer systems that have be trained using many previous chest scans to help identify signs of health conditions such as lung cancer.*

*Lastly, we will explore your views about the use of AI in chest diagnostics in future.*

*At the end of the interview, there will also be a chance to share anything else about your experiences and views. You can stop taking part at any time.*

- Say the interview will last approximately 45 minutes to one hour, or less, depending on how much they would like to say
- Emphasise that there are no right or wrong answers and taking part is completely voluntary, and that they are free to withdraw at any time
- Say that the interview will be audio recorded and professionally transcribed, but findings will be fully anonymised (names, places)
- Thank for providing consent ahead of interview, or take verbal consent – check still happy to participate
- Turn recorder on
- Check if they are a patient or carer (if not known from recruitment and eligibility)
- **inform them about NHS Trust contact they can use if they have any direct queries about their care**

***Part 1 – Experiences of diagnostic care***

| **Topic** | **Main question/s** | **Follow-up questions/prompts** |
| --- | --- | --- |
| ABOUT THEM | 1. Please could you briefly tell me: 2. If you (or someone you care for) had an X-Ray, CT scan or both? 3. When did this take place, and which hospital? | - How were you/ the person you care for referred for the scan? (eg. GP, emergency department) |
| CARE, INVESTIGATIONS AND OUTCOMES | 1. How did you find the experience? 2. *If you feel comfortable sharing -* what was your experience getting the results? 3. What happened afterwards? | - What did having a scan involve? - What information were you given, and by who? How did you find the communication between services? - How long did the X-ray and/or CT scan take? Time in between referral and having the X-ray and/or CT scan done? - If previous experience of a chest X-ray/CT scan – notice any differences in the process? - Did you have any concerns and/or worries? - What helped when going for your scan/ getting your results? - How long did it take to get your results? - Were the next steps of your care clear? Were they easy to understand? - How quickly did these next steps happen? |

***Introduction to part 2 (can repeat this if needed):***

As discussed in the beginning of the interview, in this second part we will ask you about AI and your views on it being used in chest diagnostics.

When we talk about ‘artificial intelligence’ or ‘AI’ what we mean by this are computer systems that have been trained using many previous chest scans to help identify signs of health conditions such as lung cancer. For example, AI can be used to look at X-ray images to help specialist healthcare staff like radiographers make decisions. This might help ensure a patient with an urgent condition is checked more quickly. It is important to say that when AI is used for this purpose, AI does not make decisions alone; it is used as a tool to help healthcare staff (e.g., radiographers) and there is always a clinician making the final decision about a patient’s care. There is evidence to show that AI tools may have a similar accuracy to a human radiographer (i.e. there is a similar level of error). These AI tools are used in the NHS and the safety of the tools are regularly checked.

If you have any concerns about this, we have resources we can provide for you at the end of the interview.

The next few questions will ask you what you think about the way AI is currently being used in chest diagnostics.

***Part 2*** **– *Views on the current use of AI in chest diagnostics***

| **Topic** | **Main question/s** | **Follow-up questions/prompts** |
| --- | --- | --- |
| AI IN CHEST DIAGNOSTICS | 1. Have you heard about AI being used in chest diagnostics? | - How did you hear about this? (family & friends, hospital, work etc)? - How much information was given to you? And what did you think about it? - Did you have any initial thoughts on how AI is being used in chest diagnostics? *(explore any initial assumptions or misconceptions)* |
| VIEWS ON **CURRENT AI** **USE** TO SUPPORT DIAGNOSTICS | 1. What, if anything, were you told about AI being used to look at your X-ray and/or CT scans? | If they have been told (though may be some overlap):   - How were you told about AI being used? - When were you told about AI being used? - Do you think you had enough information? - What else would you have liked to know? - How much information do you think you should have been told? (e.g., where your information would be sent, how it would be processed, and which staff would be involved?)   Would you have liked the choice of opting in or out of AI being used?  If they haven’t been told (though may be some overlap):   - Do you think that you should be told about AI being used, and why? - When should you be told about AI being used? - How much information do you think you should have been told about AI being used? - Would you have liked the choice of opting in or out of AI being used?   **Refer interviewee to NHS Trust contact if they have additional questions* |
|  | 1. What do you think about specialist staff (like radiologists, radiographers) looking at an X-ray or CT scan with AI input (e.g., to highlight signs of cancer or other health conditions, or indicate that the scan is normal) **to help when making their decisions?** | - What do you think is good or bad about AI being used like this? For patients, clinicians and NHS/wider system - Is there anything that would influence your perspective? *Prompts:*   - How the use of AI is communicated to you?   - Staff available at the hospital to review scans?   - Length of time the specialist staff reading your scan have been qualified for?   - Amount of training the staff have had to use the tool?   - How severe the condition is?   - The number and type of scans used to train the AI tool (e.g, scans on people with different conditions and backgrounds)? |
|  | 1. What do you think about AI being used **to prioritise X-rays or CT scans** to be reviewed by specialist staff (e.g., if the AI suggests there might be signs of cancer or other health conditions then these cases are checked by specialist staff more quickly)? | - What do you think is good or bad about AI being used like this? For patients, clinicians and NHS/wider system - Is there anything that would influence your perspective? *Prompt:*   - How the use of AI is communicated to you?   - Staff available at the hospital to review scans?   - Length of time the specialist staff reading your scan have been qualified for?   - Amount of training the staff have had to use the tool?   - How bad the condition is?   - The number and type of scans used to train the AI tool (e.g., scans on people with different conditions and backgrounds)? |
| ***Introduction to example scenarios***  We’re now going to look at some example situations that could happen when AI is used to help review chest X-rays.  Before we go through these examples, it’s worth knowing that:   - there is some evidence to show that AI tools have a similar accuracy to a human radiographer (i.e. there is a similar level of error) - the safety of the AI tools are regularly checked - situations like these can also happen now, even when AI isn’t used. For example, it’s common for some people to have extra tests that then show nothing serious, and sometimes important findings are only spotted a little later. In these examples, the AI is simply there to help the healthcare team decide which X-rays to look at sooner — but a trained clinician will always make the final decision about your care.   In each example, I’ll explain what happens and then ask you how you might feel in that situation and what might influence your view. | | |
| VIEWS ON POTENTIAL **OUTCOMES** OF AI USE  *[True positive;*  *False positive;*  *False negative;*  *True negative]* | 1. **OPTIONAL SCENARIO 1** (TRUE POSITIVE) The AI tool does a first reading of a patient’s X-ray, and highlights possible signs of cancer. The X-ray is then prioritised, and looked at by specialist staff sooner. The specialist agrees with the AI and recommends a CT scan, which gives more detailed information and confirms findings suspicious of cancer. AI has helped prioritise the initial specialist review so that the patient *could* then get diagnosed and treated more quickly. | - How would you feel about your scan being prioritised in this situation? - How much trust would you have in the use of AI in chest diagnostics, in this situation? (on a scale of 1-5, 5 being full trust) - Would you want to be told that AI had been used to help the specialist review your scan, or would that not be important to you as long as a specialist was making the final decision?   **OPTIONAL SCENARIO** |
|  | 1. **SCENARIO 2** (FALSE POSITIVE) The AI tool does a first reading of a patient’s X-ray, and it highlights possible signs of cancer. The X-ray is then prioritised to be looked at by specialist staff sooner. The specialist agrees with the AI and recommends a CT scan, which gives more detailed information. The CT scan suggests the patient has no signs of cancer or other health conditions, and the patient is reassured by their clinician (eg GP). | - How would you feel about having an additional CT scan in this situation? - How much trust would you have in the use of AI in chest diagnostics, in this situation? (on a scale of 1-5, 5 being full trust) - If we told you that going for additional scans happens quite often in standard NHS care without AI, would this change your view?* - Would you want to be told that AI had been used to help the specialist review your scan, or would that not be important to you as long as a specialist was making the final decision?   **Note: In NHS cancer pathways, it is common for patients to have further tests that do not confirm cancer — e.g. only about 3% of patients referred on a suspected lung cancer pathway are found to have the disease. This process helps ensure that potential cancers are not missed.* |
|  | 1. **SCENARIO 3** (FALSE NEGATIVE) The AI tool does a first reading of a patient’s X-ray, and it finds no signs of cancer or other health conditions. This means that it is not flagged for urgent specialist staff review, and is looked at by staff in the usual timeframe. When specialist staff look at the X-ray, they identify suspected cancer and recommend a CT scan, which confirms findings suspicious for cancer. The patient then goes for more tests to confirm the cancer diagnosis. In this case, AI missed the opportunity to fast-track the initial specialist review. | - How would you feel about your scan not being prioritised in this situation? - How long a delay to the clinician’s report would be acceptable to you? (e.g. up to 3 days, 1 week, 2 weeks?) - How much trust would you have in the use of AI in chest diagnostics, in this situation? (on a scale of 1-5, 5 being full trust) - Would you want to be told that AI had been used to help the specialist review your scan, or would that not be important to you as long as a specialist was making the final decision? |
|  | 1. **OPTIONAL SCENARIO 4** (TRUE NEGATIVE) The AI tool reads a patient’s X-ray, and it finds no signs of cancer or other health conditions. This means that it is not flagged for urgent specialist staff review, and is looked at by staff in the usual timeframe. When specialist staff look at the X-ray, they agree there are no suspicious findings and issue a radiology report. The report explains the X-ray is normal but advises the clinician to review the patient in 6 weeks and consider further investigation if symptoms persist. This might take longer to report than the X-rays AI has prioritised, but is still checked within the hospitals target reporting times for normal X-rays. | - How would you feel about your scan not being prioritised in this situation? (Would it matter to you if your results took slightly longer because other, more urgent scans were prioritised?) - How much trust would you have in the use of AI in chest diagnostics, in this situation? (on a scale of 1-5, 5 being full trust) - Would you want to be told that AI had been used to help the specialist review your scan, or would that not be important to you as long as a specialist was making the final decision?   **OPTIONAL SCENARIO** |
| ***Introduction to future look***  So far in this interview, we have been discussing examples of the way AI is *currently* being used in the NHS. However, in this next section we are going to ask you about a potential way AI might be used *in future*, where it may play a greater role in patient care. | | |
| VIEWS ON POTENTIAL AUTONOMOUS AI REPORTING IN FUTURE (I.E. NO HUMAN REVIEW) | 1. In future, *if the law was changed*, AI might be used to review and report a patient’s X-ray without any human involvement (ie specialist staff checking it). This would only happen if it can be proved that AI tools are as good as or better than specialist staff at reading X-rays.   Any such changes would only be made gradually and carefully in the NHS: starting with using AI tools only on X-rays that are very likely to be normal, with regular safety checks by specialist healthcare staff. | - How acceptable would this use of AI be to you? - How much confidence/trust would you have in this use of AI? - What do you think about the use of AI in this way, for different types of patients? (e.g. ethnicity, gender, age, condition) - Who should be responsible if errors occur? (e.g. hospital clinicians, suppliers, regulators, government) - What might influence your view? |
|  | 1. What might make you confident that the process is safe, and patients get high-quality care? | *Prompts if needed:*   - The doctor who referred you can request an urgent second report (by a specialist), including out of hours - Communication - information about the process provided by charities or the hospital - The number and type of scans used to train the AI tool (e.g., scans on people with different conditions and backgrounds)? - Opt-in/opt-out and consent for AI to report directly on its own - Receiving your individual AI report (e.g., on NHS app) - Being given information about how accurate and safe the AI tool is - The AI tools have been checked by NHS bodies (e.g., NICE) - AI tools being continuously checked by specialist radiologists |
| FUTURE USE | 1. What do you think about AI being used in chest diagnostics in the future? | - Do you think that AI should be used in the future, and why? - What might concern you about AI being used in the future? - What do you think is good about using AI in the future? - What do you think about your data being used to help improve AI? - How would you feel about AI tools learning to be safer and more accurate based on yours (and other peoples scans, with consent)? |
| ***Close*** | | |
| WRAP UP | 1. Is there anything else you would like to add that we haven’t discussed? |  |
| DEMOGRAPHICS | We would just like to ask you about a few personal characteristics, are you happy to do this?   - Sex - Gender - Age - Ethnicity - Language (e.g. is English their first or second language) - Education level - Employment status / occupation - Condition/s (which required the diagnosis) - Postcode / location - Level of familiarity/understanding of AI in general | *Prompts if carer:*   - Caring responsibilities (proportion of time) - Employment status - Living situation (e.g., living alone, with others) |
| DISSEMINATION LIST | Would they like to be added?   - Yes - No |  |
